# Reactivation of CTLA4-expressing T cells accelerates resolution of lung fibrosis in a humanized mouse model

**DOI:** 10.1172/JCI181775

**Published:** 2025-03-18

**Authors:** Santosh Yadav, Muralidharan Anbalagan, Shamima Khatun, Devadharshini Prabhakaran, Yasuka Matsunaga, Justin Manges, James B. McLachlan, Joseph A. Lasky, Jay Kolls, Victor J. Thannickal

**Affiliations:** 1 John W. Deming Department of Medicine, Tulane University School of Medicine, New Orleans, Louisiana, USA.; 2 Southeast Louisiana Veterans Health Care System, New Orleans, Louisiana, USA.; 3 Department of Structural and Cellular Biology,; 4 Center for Translational Research in Infection and Inflammation, and; 5 Department of Microbiology, Tulane University School of Medicine, New Orleans, Louisiana, USA.

**Keywords:** Immunology, Pulmonology, Fibrosis, Immunotherapy, T cells

## Abstract

Tissue regenerative responses involve complex interactions between resident structural and immune cells. Recent reports indicate that accumulation of senescent cells during injury repair contributes to pathological tissue fibrosis. Using tissue-based spatial transcriptomics and proteomics, we identified upregulation of the immune checkpoint protein, cytotoxic T lymphocyte–associated protein 4 (CTLA4), on CD8^+^ T cells adjacent to regions of active fibrogenesis in human idiopathic pulmonary fibrosis and in a repetitive bleomycin lung injury murine model of persistent fibrosis. In humanized CTLA4-knockin mice, treatment with ipilimumab, an FDA-approved drug that targets CTLA4, resulted in accelerated lung epithelial regeneration and diminished fibrosis from repetitive bleomycin injury. Ipilimumab treatment resulted in the expansion of Cd3*e*^+^ T cells, diminished accumulation of senescent cells, and robust expansion of type 2 alveolar epithelial cells, facultative progenitor cells of the alveolar epithelium. Ex vivo activation of isolated CTLA4-expressing CD8^+^ cells from mice with established fibrosis resulted in enhanced cytolysis of senescent cells, suggesting that impaired immune-mediated clearance of these cells contributes to persistence of lung fibrosis in this murine model. Our studies support the concept that endogenous immune surveillance of senescent cells may be essential in promoting tissue regenerative responses that facilitate the resolution of fibrosis.

## Abstract 2

Blocking CTLA4 in immune cells may help regenerate lungs and reduce fibrosis in idiopathic pulmonary fibrosis.

## Introduction

Humans have evolved highly specialized immune systems that rely on the activity of cytotoxic T cells to eliminate irreparably damaged cells from tissues/organs. This endogenous quality control mechanism is essential for maintaining organ structure and function. In recent years, this property of cytotoxic T cells has been leveraged to design antitumor strategies in which immune checkpoint inhibitors are used to reactivate exhausted T cells for targeted killing of cancer cells (1). Opportunities to extend this endogenous immune surveillance function beyond cancer are only beginning to be explored.

Fibrosis of various organs in humans typically originates from regenerative failure or delayed repair of damaged epithelium that triggers immune and stromal tissue responses (2, 3). Cell-cell interactions within this dynamic tissue microenvironment determine the outcome of the repair-regenerative response. Recent studies have implicated the persistence of senescent cells in nonresolving fibrosis (4–8), while its transient emergence may be proregenerative (9–11). A failure in immune surveillance of senescent cells may result in the pathological conversion from proregenerative to profibrotic outcomes. Persistence of senescent cells may, in turn, create an immunosuppressive microenvironment that leads to T cell exhaustion, a process that is well appreciated in cancer progression (12–14).

Despite interest in developing senolytic agents for treating persistent/progressive fibrotic disorders (5, 15, 16), the contributions of endogenous immune-mediated cytolysis and clearance of senescent cells to fibrosis resolution remain unclear. In this report, we demonstrate that the immune checkpoint protein, cytotoxic T lymphocyte–associated protein 4 (CTLA4), was markedly upregulated in the lungs of humans with idiopathic pulmonary fibrosis (IPF). Using a humanized CTLA4 murine model with repetitive genotoxic injury-provoked fibrosis, we demonstrate that targeting CTLA4 accelerated epithelial regeneration capacity and ameliorated fibrosis.

## Results

### The immune checkpoint protein, CTLA4, is upregulated on T cells within regions of active fibrogenesis in human IPF.

**Figure 1:**
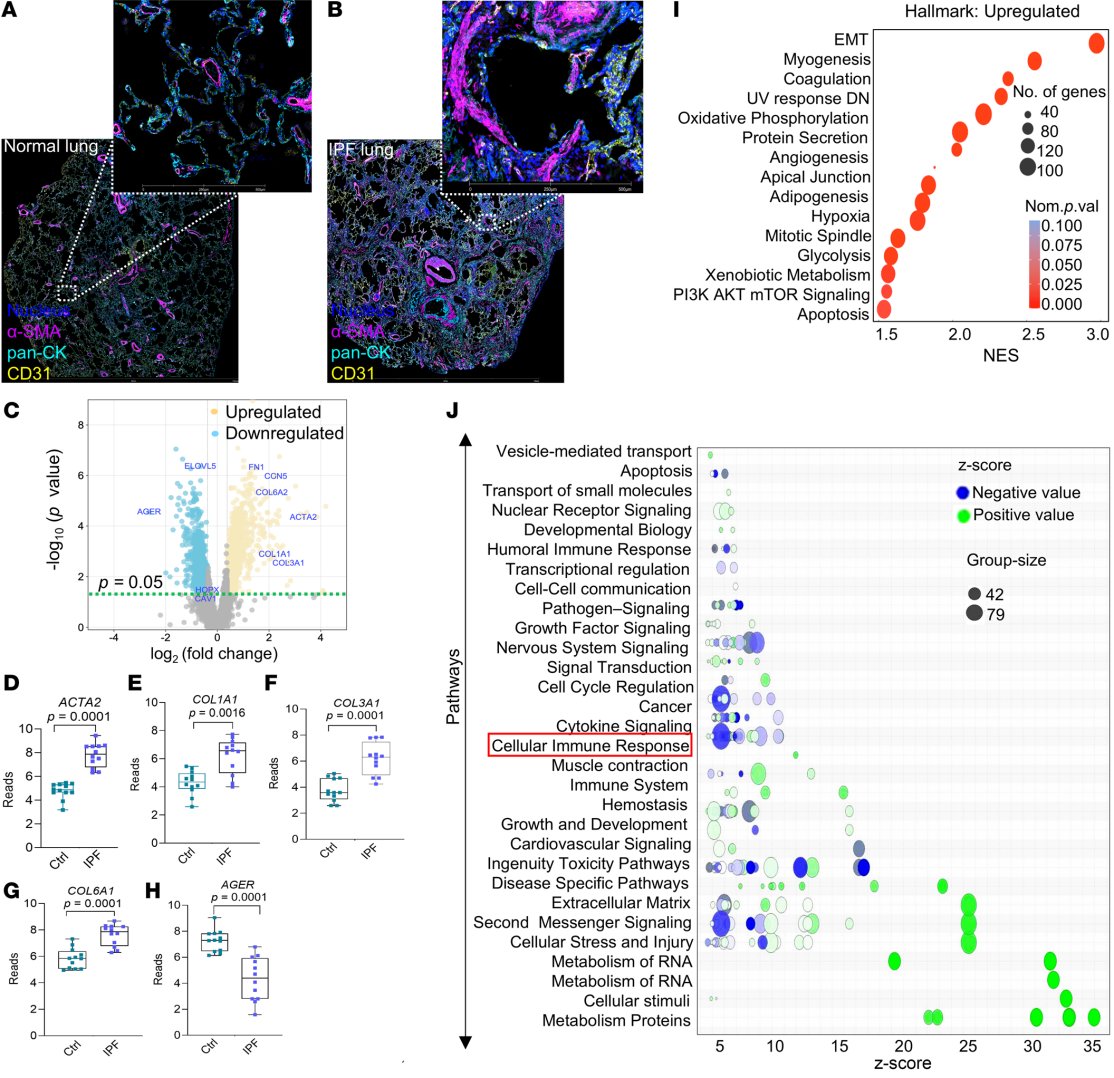
Spatial transcriptomics of IPF fibroblastic foci suggests suppression of cellular immune responses. (**A** and **B**) Spatial images of lung tissue sections from patients with IPF and healthy individuals acting as controls were acquired by digital spatial profiling (GeoMx) using fluorescence of pan-CK (cyan), CD31 (yellow), α-SMA (magenta), and DAPI (blue). Scale bar: 5 mm (**A** and **B**), 500 μm (higher-magnification images). (**C**) Volcano plot of differentially expressed genes with an FDR threshold of –log_10_ (P < 0.05). (**D**–**H**) Bar plots showing read counts for *ACTA2*, *COL1A1*, *COL3A1*, *COL6A1*, and *AGER*. (**I**) GSEA Hallmark pathway analysis of differentially expressed genes, comparing IPF vs. control (*n* = 12 ROIs each; *P* < 0.05; normalized enrichment score ≥ 1.5). (**J**) IPA analysis was performed on differentially expressed genes of healthy individuals acting as controls and patients with IPF. The cellular immune response that is downregulated is highlighted in red. Data are shown as mean ± SEM. Statistical differences in **D**–**H** were tested using a paired 2-tailed Student’s *t* test.

Spatial transcriptomics and proteomics provide new insights into cell-cell interactions within tissue niches of normal and diseased organs (17). We conducted spatial transcriptomics and proteomics in the lung tissues of individuals with the progressive and fatal lung disorder, IPF. In this disease process, active fibrogenesis occurs within morphological lesions referred to as “fibroblastic foci,” marked by the presence of α-smooth muscle actin–expressing (α-SMA–expressing) myofibroblasts that are normally not present in alveoli of nonfibrotic postnatal adult lung where this marker is primarily restricted to smooth muscle cells surrounding more proximal airways and muscularized blood vessels. To accurately identify fibroblastic foci, we stained IPF lung tissues for α-SMA, pan-cytokeratin (pan-CK, a marker of lung epithelium), and CD31 (a marker of vascular endothelium) (Figure 1, A and B). Twelve regions of interest (ROIs) capturing fibroblastic foci within lung sections of 2 different participants with IPF and 12 ROIs from 2 age-matched individuals acting as controls were analyzed using a Digital Spatial Profiler (DSP) Platform (nanoString GeoMx, Supplemental Figure 1; supplemental material available online with this article; https://doi.org/10.1172/JCI181775DS1). We assayed 18,676 genes from the GeoMx transcriptomics atlas within these 24 ROIs, and 9,101 of the genes were detected in ≥1% of segments. Differential expressions of these genes based on a log_2_ fold change cutoff of 1.5 and *P* < 0.05 showed an upregulation of classical profibrotic genes, including α-SMA (*ACTA2*), collagen 1A1 (*COL1A1*), collagen 3A1 (*COL3A1*), and collagen 6A2 (*COL6A2*), in IPF versus control ROIs (Figure 1, C–G). In contrast, the alveolar type 1 (AT1) epithelial marker gene, advanced glycosylation end product–specific receptor (*AGER*), was markedly downregulated (Figure 1H); other AT1 markers, HOP homeobox (*HOPX*; 0.39 log_2_ fold, *P* = 0.052) and caveolin 1 (*CAV1*; 0.45 log_2_ fold, *P* = 0.054), were downregulated but did not reach statistical significance. Gene set enrichment analysis (GSEA) revealed several GSEA-Hallmark pathways associated with fibrosis that were found to be upregulated in IPF ROIs (*P* < 0.05, normalized enrichment score ≥ 1.5; Figure 1I). Interestingly, Ingenuity Pathway Analysis (IPA) of differentially expressed genes between control and IPF lungs revealed decreased cellular immune response (Figure 1J; full list of genes in this IPA, Supplemental Table 1).

**Figure 2:**
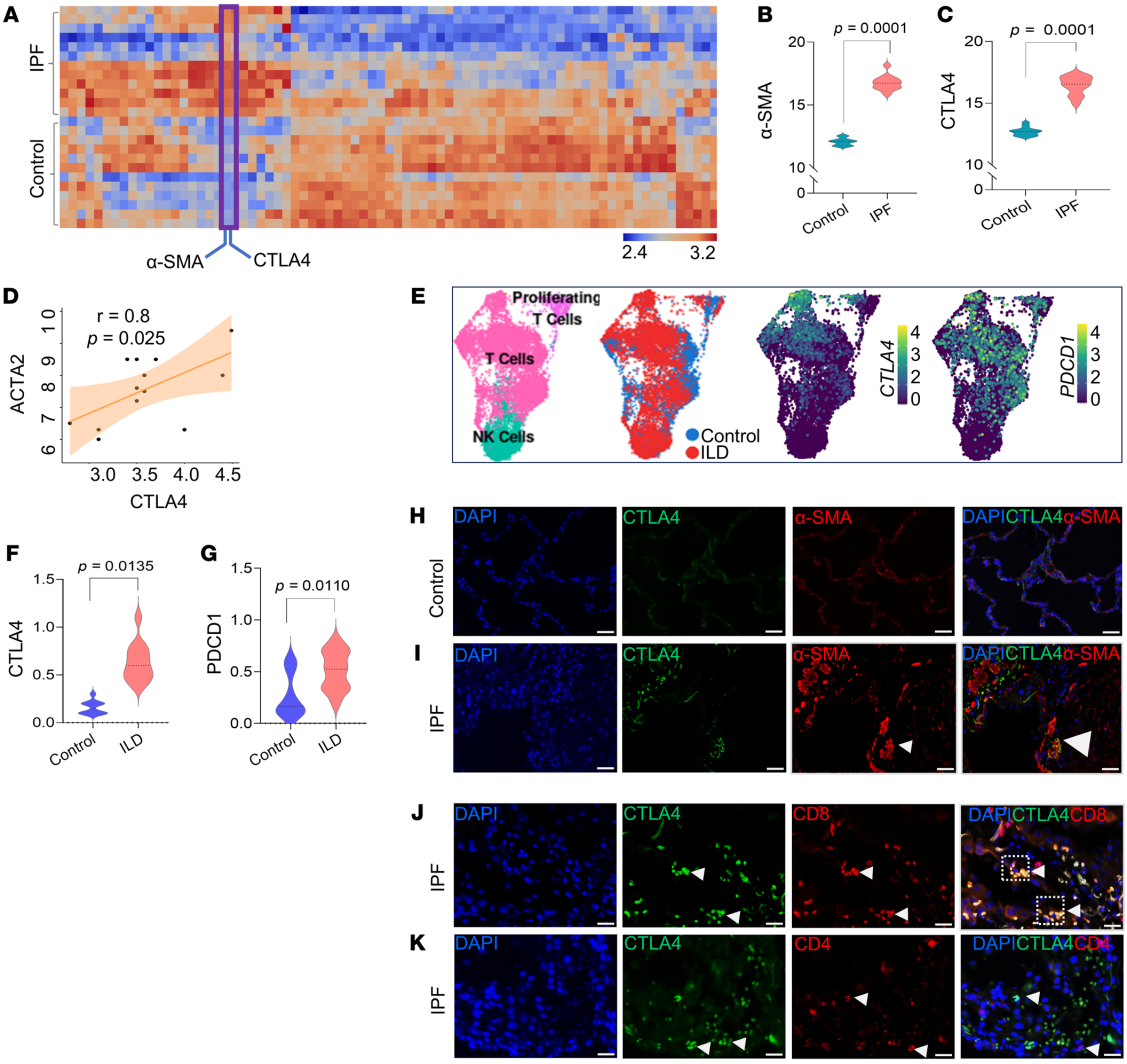
The immune checkpoint inhibitor CTLA4 is upregulated within fibrotic niches in the lungs of patients with IPF. (**A**) Unsupervised hierarchically clustered heatmap of protein counts for IPF (*n* = 12 ROIs) and control (*n* = 12 ROIs). Protein profiling was performed with a 79-plex oligonucleotide–antibody cocktail for 24 ROIs. (**B** and **C**) Violin plots showing normalized read counts of α-SMA and CTLA4. Statistical comparisons are between reads from healthy individuals acting as controls (*n* = 12 ROIs) and patients with IPF (*n* = 12 ROIs). (**D**) Correlation of *CTLA4* and *ACTA2*. Statistical comparisons are between reads from healthy individuals acting as controls (*n* = 12 ROIs) and patients with IPF (*n* = 12 ROIs). (**E**) UMAP of *CTLA4* and *PDCD1* expression in T cells from the Banovich/Kropski dataset in the IPF Cell Atlas (https://www.ipfcellatlas.com/). (**F** and **G**) *CTLA4* and *PDCD1* expression in T cells (patients with interstitial lung diseases [ILD] vs. healthy individuals). (**H** and **I**) Representative immunostaining images of lung tissues from healthy individuals acting as controls and patients with IPF (CTLA4, green; α-SMA, red; and DAPI, blue). Arrowheads indicate fibroblastic foci. Scale bar: 50 μm. (**J** and **K**) Expression of CTLA4 on CD8^+^ or CD4^+^ cells in lung tissues from patients with IPF (CTLA4, green; CD8^+^ T cell, red; CD4^+^T cell, red; and DAPI, blue). Arrowheads indicate colocalization of CTLA4 with CD8^+^ cells, but not CD4^+^ cells. Scale bar: 50 μm. Data are shown as mean ± SEM. Statistical differences in **B**, **C**, **F**, and **G** were tested using a paired 2-tailed Student’s *t* test.

Next, to gain further insights into immune dysfunction in IPF, we employed a proteomics immune panel (nanoString GeoMx) encompassing 79 protein targets and 7 protein modules: human immune cell profiling protein core, human immune activation status protein module, human immune cell typing protein module, human pan-tumor protein module, human PI3K/AKT signaling protein module, human cell death protein module, and human MAPK signaling protein module. Spatial profiling of these proteins across the same IPF and control tissues employed for spatial transcriptomics confirmed a high expression of CTLA4 protein in ROIs enriched in α-SMA (Figure 2, A–D; see Supplemental Table 2 for a complete list of all 79 proteins). To determine if the finding of upregulated expression of CTLA4 was more broadly applicable to human lung fibrotic disorders, we queried a publicly available single-cell RNA-Seq (scRNA-Seq) dataset (IPF Cell Atlas; https://www.ipfcellatlas.com/); this confirmed that CTLA4 is highly expressed in pulmonary fibrosis, preferentially within the T cell population, in a cohort of 20 patients with pulmonary fibrosis, including 12 with IPF and 10 individuals acting as controls, representing 114,396 total cells (Figure 2, E–G). We examined the expression of another immune checkpoint protein, programmed cell death protein 1 (PDCD1), in the same IPF Cell Atlas dataset; PDCD1 expression levels were similar between the IPF and control groups (Figure 2G) and more ubiquitously expressed in T cells (Figure 2G). In our spatial proteomics dataset, the expression of PD-1 protein (encoded by PDCD1) was significantly increased; however, this increase was markedly less (1.8-fold on the linear scale; 0.89 log_2_) than the observed increase in CTLA4 protein (32-fold on the linear scale; 5.01 log_2_) (Supplemental Table 2).

**Figure 3:**
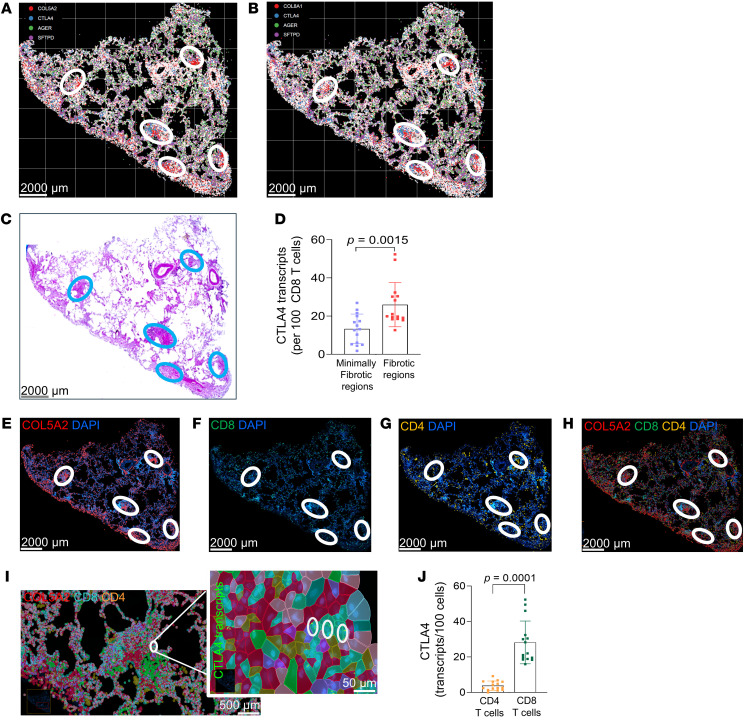
CTLA4 is upregulated on CD8^+^ T cells adjacent to COL5A2-expressing cells in fibrotic regions of IPF lungs. (**A**) Xenium spatial plot showing expression of *COL5A2*, *CTLA4*, *AGER*, and *SFTPD* in fibrotic regions. White circles indicate fibroblastic foci expressing *COL5A2* (red) and adjacent cells expressing *CTLA4*. Scale bar: 2,000 μm. (**B**) Xenium spatial plot showing expression of *COL8A1*, *CTLA4*, *AGER*, and *SFTPD* in fibrotic regions. Scale bar: 2,000 μm. (**C**) Post-Xenium H&E staining of IPF lung tissue. Scale bar: 2,000 μm. (**D**) *CTLA4* transcript levels in less fibrotic and fibrotic ROIs. Data are from 3 IPF lungs, with 5 fibrotic and less fibrotic regions analyzed per IPF lung. (**E**–**H**) Xenium spatial plot showing expression of *COL5A2*, *CD8*, and *CD4* and overlay in IPF lung. Scale bar: 2,000 μm. (**I**) Xenium spatial plot showing expression of *COL5A2*, *CD8*, and *CD4* with a high-magnification image showing CTLA4 transcript expression on CD8^+^ T cells. (**J**) CTLA4 transcript numbers in CD4^+^ T cells vs. CD8^+^ T cells in IPF lungs. Scale bar: 500 μm; 50 μm (high-magnification image). Data are shown as mean ± SEM. Statistical differences in **D** and **J** were tested using a paired 2-tailed Student’s *t* test.

Based on the expression pattern of CTLA4, we explored the spatial relationships of T cells within fibroblastic foci by immunofluorescence staining of individuals with IPF and individuals acting as controls. CTLA4^+^ cells were found to be localized near or adjacent to α-SMA–expressing myofibroblasts in IPF, while these cells were relatively absent around normal α-SMA–expressing smooth muscle cells lining blood vessels or airways in individuals acting as controls (Figure 2, H and I). Within fibroblastic foci of IPF lungs, CD8^+^ T cells represented the predominant T cell subset expressing CTLA4 when compared with CD4^+^ T cells (Figure 2, J and K). To further explore the spatial relationships of these cells in IPF lungs, we performed spatial transcriptomics using the 10x Genomics Xenium transcriptomics platform designed for the human lung. In 3 IPF lung samples, we identified a total of 62,485 cells, containing 55,928,562 transcripts from a 289 gene panel. Using the Xenium-annotated myofibroblast markers, *COL5A2* and *COL8A1* (Figure 3, A and B), we were able to distinctly identify fibroblastic foci that localized to previously identified regions by histopathological staining (Figure 3C). To determine the expression of *CTLA4* at single-cell resolution in CD8^+^ T cells, we quantitated transcript levels of *CTLA4* in this cell population and compared relative levels of expression in fibrotic regions (enriched for *COL5A2*) compared with less fibrotic regions (enriched for *AGER*); this analysis showed significantly higher *CTLA4* levels in CD8^+^ T cells localized to fibroblastic foci than in regions of the IPF lung without distinct foci (Figure 3D; bar graph representing fibrotic regions and minimally fibrotic regions, 5 regions each from 3 different IPF lungs; *n* = 15 per group).

Furthermore, we characterized the gene expression patterns of CTLA4 in specific T cell subpopulations using Xenium-based spatial transcriptomics. Interestingly, we found that fibroblastic foci appear to be markedly enriched for CD8 gene expression, while CD4 was more uniformly expressed throughout the IPF lung (Figure 3, E–H). Importantly, within fibroblastic foci, CTLA4 was preferentially upregulated in CD8-expressing cells in comparison to CD4 cells (Figure 3, I and J), 5 regions each from 3 different IPF lungs; *n* = 15 per group). These data suggest a potential role for cell-cell crosstalk between CTLA4^+^ expressing CD8^+^ T cells and myofibroblasts within active fibrotic niches of human IPF lungs.

### CTLA4 checkpoint blockade induces alveolar type 2 cell regeneration and resolves fibrosis.

**Figure 4:**
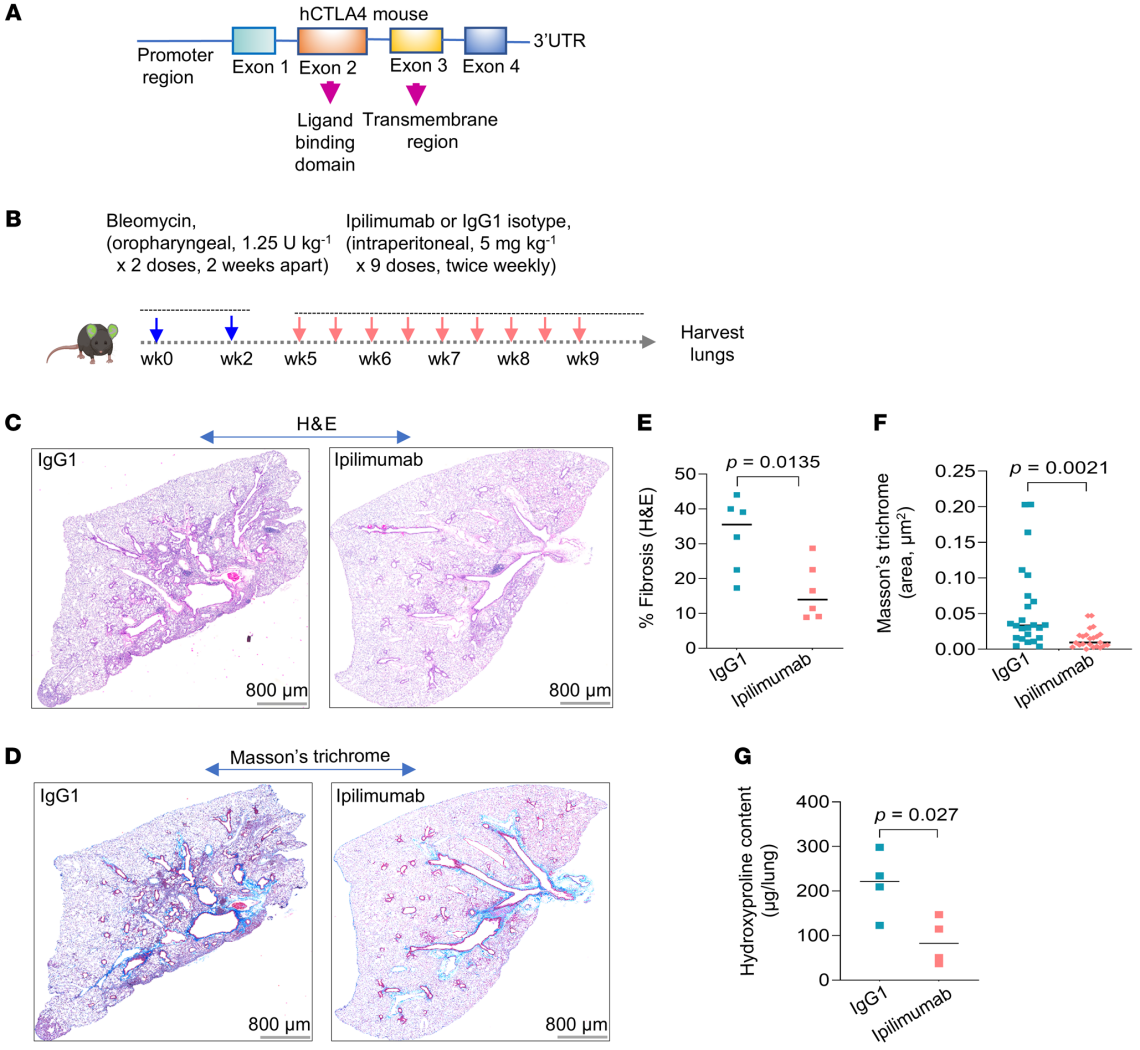
CTLA4 blockade in a mouse model of persistent lung fibrosis ameliorates fibrosis. (**A**) Schematic representation of the humanized CTLA4-knockin mouse carrying the extracellular domain of *CTLA4* gene. (**B**) Experimental outline of humanized CTLA4 mice subjected to repetitive bleomycin injury–induced pulmonary fibrosis via oropharyngeal administration of bleomycin (1.25 U per kg body weight) in 2 doses, 14 days apart. On day 21 after the second dose, mice were treated intraperitoneally with either control IgG1 isotype or the anti-CTLA4 monoclonal antibody ipilimumab at doses of 5 mg per kg body weight twice per week for 4.5 weeks (total of 9 doses). (**C** and **D**) Representative images of H&E- (**C**) and Masson’s trichrome–stained (**D**) whole lung sections from bleomycin-induced mice treated with either IgG1 isotype control or ipilimumab. Scale bar: 800 μm. (**E**) Quantitation of H&E staining in the IgG1 isotype control and ipilimumab groups. Images of H&E staining were quantified using QuPath software. Data represent IgG1 isotype control and ipilimumab (*n* = 6 in each group). (**F**) Quantitation of Masson’s trichrome staining in the IgG1 isotype control or ipilimumab groups. Collagen containing areas were identified with machine-learning algorithms using inForm automated image analysis software built into the PhenoImager Fusion (Akoya Biosciences). Data represent IgG1 isotype control and ipilimumab groups (*n* = 6 per group). (**G**) Whole-lung hydroxyproline content (μg/lung) in IgG1 isotype control and ipilimumab treated groups (*n* = 4 per group). Data are shown as mean ± SEM. Statistical differences in **E**–**G** were tested using a paired 2-tailed Student’s *t* test.

CTLA4 has been linked to deficient T cell responses in cancer (18), but its role in fibrotic disorders remains unclear. To determine a potential role for this immune checkpoint protein in lung fibrosis, we employed a humanized mouse model carrying exon 2 and 3 of the human *CTLA4* gene (*hCTLA4* mice; C57BL/6 background) encoding an epitope containing its extracellular ligand binding and transmembrane domains (Figure 4A); this epitope is known to bind the FDA-approved anti-CTLA4 monoclonal antibody, ipilimumab (19). Mice were treated with 2 doses of bleomycin (1.25 U per kg body weight) 14 days apart to induce a more sustained fibrotic response (20). To more specifically target the fibrotic (vs. inflammatory) response, we started treatment 21 days after the second bleomycin dose with either ipilimumab or control isotype IgG1 (5 mg kg per kg body weight twice per week for 9 doses) (Figure 4B). Ipilimumab-treated mice showed diminished fibrosis, as evidenced by H&E staining (Figure 4C) and Masson’s trichrome staining (Figure 4D), QuPath quantitation of H&E staining (Figure 4E), quantitative Masson’s trichrome staining (Figure 4F), and total lung hydroxyproline levels (Figure 4G). Furthermore, gene expression levels of *acta2* and *col1A1* were reduced in lungs of mice treated with ipilimumab (Supplemental Figure 2).

**Figure 5:**
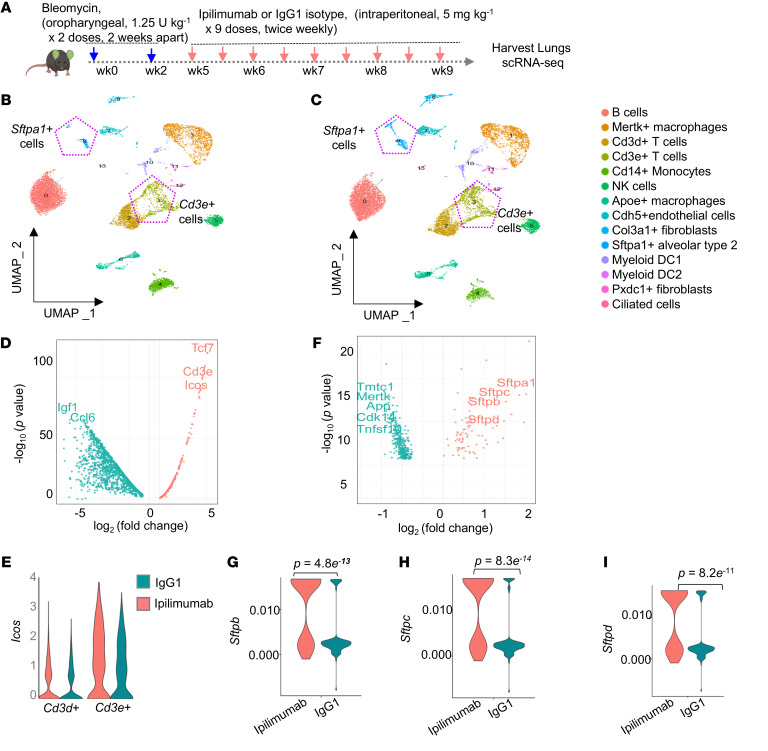
Fibrosis resolution in mice with CTLA4 blockade is associated with activation of T cell subsets and expansion of alveolar type 2 cells. (**A**) Experimental outline of humanized CTLA4 mice subjected to repetitive bleomycin injury–induced pulmonary fibrosis via oropharyngeal administration of bleomycin (1.25 U per kg body weight) in 2 doses,14 days apart. On day 21 after the second dose, mice were treated intraperitoneally with either control IgG1 isotype or the anti-CTLA4 monoclonal antibody ipilimumab at doses of 5 mg per kg body weight twice per week for 4.5 weeks (total of 9 doses). (**B** and **C**) UMAP of whole-lung cells from the IgG1 isotype control and ipilimumab groups. Cell clusters identified by scRNA-Seq are colored by cell type. The UMAP indicating the sftpa1^+^ cell cluster is highlighted with a hexagon. (**D**) Volcano plot showing differential gene expression in T cell clusters of the scRNA-Seq IgG1 isotype control and ipilimumab dataset. A total of 99 differentially expressed genes were identified [log_2_(fold change) > 0.3 and FDR-adjusted *P* < 0.05]. (**E**) Violin plot showing gene expression of *Icos* in the Cd3δ^+^ and Cd3ε^+^ T cell clusters of the scRNA-Seq data for whole lung cells from the IgG1 isotype control or ipilimumab groups. (**F**) Volcano plot showing differential gene expression in the sftpa1^+^ cell cluster of the scRNA-Seq dataset for the IgG1 isotype control and ipilimumab groups. A total of 399 differentially expressed genes were identified [log_2_(fold change) > 0.3, FDR-adjusted *P* < 0.05]. (**G**–**I**) Violin plots showing AT2 cell expression of the marker genes *sftpb*, *sftpc*, and *sftpd* in the in sftpa1^+^ cell cluster.

**Figure 6:**
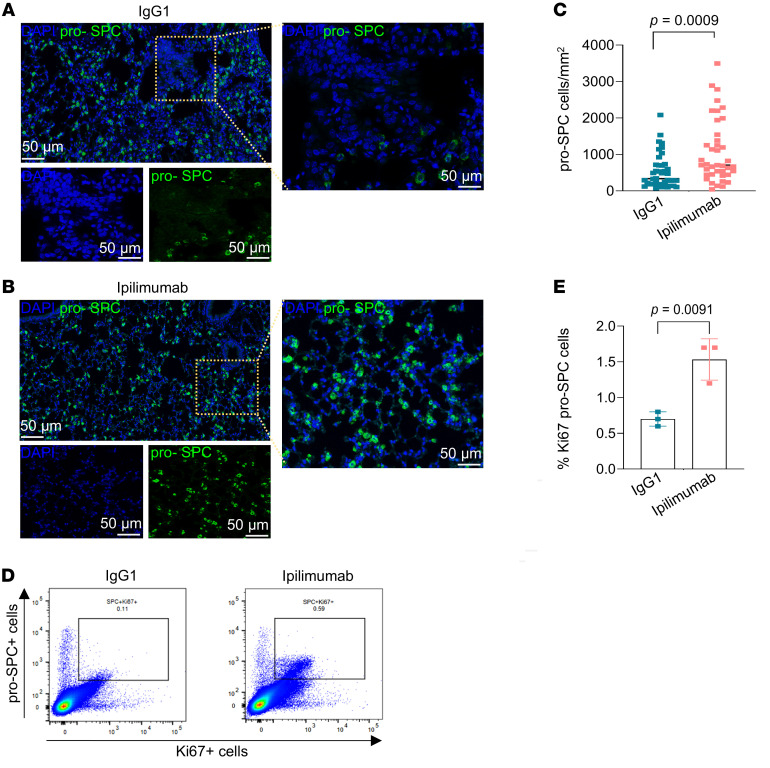
Senescent cell accumulation is reduced in mice with resolving fibrosis. (**A** and **B**) Representative images of pro-SPC^+^ cells (green) and DAPI (blue) in IgG1 isotype control and ipilimumab groups. Scale bar: 50 μm. (**C**) Phenotyping of pro-SPC^+^ cells. Images were processed with machine-learning algorithms in inForm automated image analysis software built in PhenoImager Fusion. Spatial analysis of pro-SPC^+^ cells in IgG1 isotype control and ipilimumab groups (*n* = 30 per group, representing 6 ROIs from 5 mice per group). (**D**) Representative flow cytometry plots of Ki67^+^SPC cells in IgG1 and ipilimumab groups. (**E**) The percentage of SPC and Ki67^+^ cells in IgG1 isotype control and ipilimumab groups (*n* = 3 mice per group). Data are shown as mean ± SEM. Statistical differences in **C** and **E** were tested using a paired 2-tailed Student’s *t* test.

To gain insights into the mechanisms by which CTLA4 immune checkpoint blockade mediates its antifibrotic effects, we performed scRNA-Seq on lungs of mice treated with ipilimumab and control isotype IgG1 (Figure 5A). Consistent with its targeted mechanism of action, ipilimumab treatment resulted in the expansion of T cells, specifically Cd3*e*^+^ T cells (Figure 5, B and C); importantly, the T cell activation/differentiation marker genes, *Icos* and *Tcf7*, were markedly increased in T cells (Figure 5D), with preferential upregulation in Cd3*e*^+^ T cells, in comparison to Cd3*d*^+^ T cells (Figure 5E). Flow cytometric analyses of collagenase-digested lungs at the same time-point showed an increase in both the percentage of CD8^+^ cells and the subpopulation expressing CTLA4 following ipilimumab treatment (Supplemental Figure 3). In concert with these changes in T cell subpopulations, a remarkable increase in the number and percentage of alveolar epithelial type 2 (AT2; marked by the *Sftpa1* gene) cells, facultative stem cells of the distal lung epithelium, were noted in the ipilimumab-treated group (Figure 5, B and C, and Supplemental Figure 4). In this Sftpa1^+^ AT2 cell cluster, we confirmed increased gene expression of other surfactant protein (Sftp) coding genes, *Sftpb*, *Sftpc*, and *Sftpd* (Figure 5, F–I). A total of 399 genes were differentially expressed between the ipilimumab and control isotype IgG1 group in the Sftpa1^+^ AT2 cell cluster (see Supplemental Table 3 for a complete list of genes). The expansion of AT2 cells in the ipilimumab-treated group versus the control isotype IgG1 group was confirmed by multiplex single-cell spatial phenotyping (Figure 6, A–C). This involved analyzing pro–surfactant protein C–expressing (pro-SPC-expressing) cells in whole-lung tissue sections, along with α-SMA, CD8, and CTLA4 staining to visualize distribution of epithelial cells, myofibroblasts, and CD8^+^ T cells and the expression of CTLA4. These studies confirmed the proximity of CTLA4-expressing CD8^+^ T cells in regions of active fibrosis enriched in α-SMA–expressing myofibroblasts and overall reduced population of pro-SPC–expressing AT2 cells in bleomycin-injured mice, while this association of “exhausted” CD8^+^ T cells with myofibroblasts was reduced in ipilimumab-treated mice in association with an expansion of AT2 cells (Supplemental Figure 5). Furthermore, we assayed the proliferative state of AT2 cells at the end of the ipilimumab treatment period; flow cytometric analysis of lung cells coexpressing SPC and Ki67 demonstrated a significantly higher percentage of Ki67^+^ AT2 cells in the ipilimumab group compared with the control IgG group (Figure 6, D and E). Together, these data indicate that targeting CTLA4 mediates activation and expansion of CD8^+^ T cells in parallel with robust epithelial regenerative responses that enhance fibrosis resolution following lung injury.

### Ipilimumab reduces the accumulation of p16^Ink4a^-expressing senescent cells in vivo and potentiates CD8^+^ T cell cytotoxic responses ex vivo following fibrogenic lung injury.

**Figure 7:**
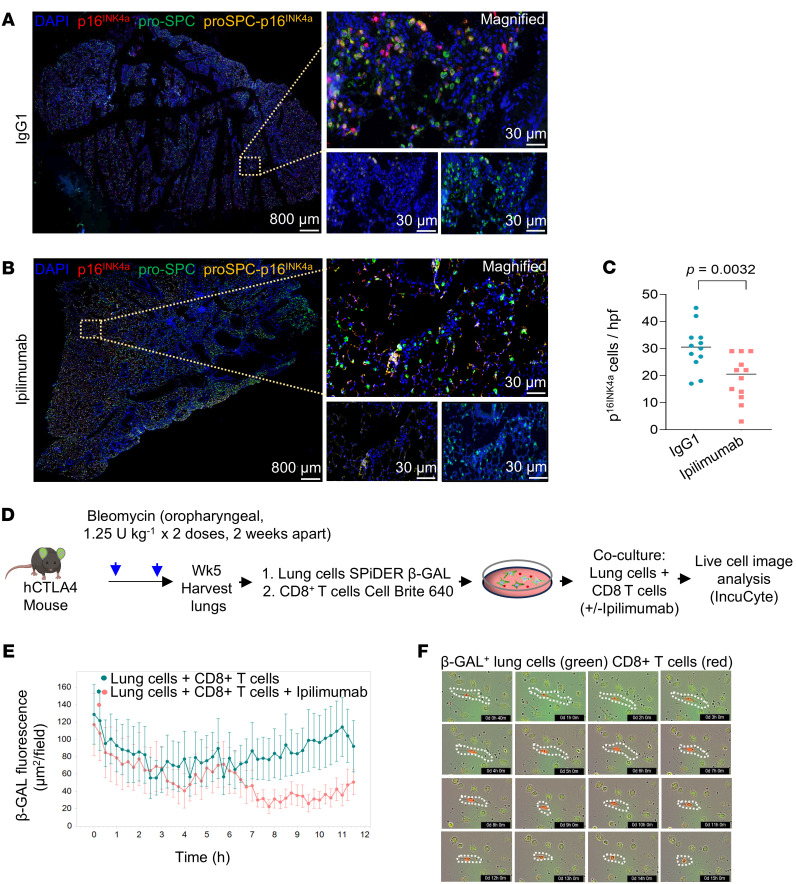
CTLA4 blockade decreases the accumulation of p16^INK4a^-expressing cells in vivo and mediates killing of senescent cells ex vivo. (**A** and **B**) Immunohistochemistry staining of p16^INK4a^ in lung tissue sections from the 2 groups receiving either IgG1 isotype control or ipilimumab. Scale bar: 800 μm (left); 30 μm (right). (**C**) Quantitation of p16^INK4a+^ cells observed in high-power field (hpf) images of the IgG1 isotype control and ipilimumab (*n* = 4 in each group). (**D**) Schematic representation of ex vivo studies of CD8^+^ T cell–mediated killing. (**E**) Lung cells isolated from bleomycin-injured mice were stained with SPiDER-β-GAL and cocultured with CD8^+^ T cells (2:1 ratio) isolated from the same mice for 12 hours with or without ipilimumab (0.05 μg/mL). Real-time detection of fluorescent area of β-GAL^+^ cells/image (*n* = 16 images per condition at each time point indicated). (**F**) Images of CD8^+^ T cell–mediated killing of β-GAL^+^ lung cells. Images were taken using live-cell imaging with the IncuCyte system. The CD8^+^ T cells were labeled with Cellbright 640 (Biotium) (red) to visualize their interaction with β-GAL^+^ (green) lung cells. Data are shown as mean ± SEM. Statistical differences in **C** were tested using a paired 2-tailed Student’s *t* test.

Cellular senescence is increasingly implicated in the etiology of age-related diseases, including organ fibrosis (21, 22). p16^Ink4a^-expressing cells, both epithelial and mesenchymal, have been found to be increased in lung samples of individuals with IPF (4, 5, 23), and senolytic clearance by pharmacologic approaches have been proposed as a potential treatment for IPF (24). To determine whether CTLA4 checkpoint blockade functions as an endogenous immune mechanism for clearance of senescent cells, we compared the number of p16^Ink4a^-expressing cells in lung tissues of mice treated with ipilimumab and control IgG1. Significantly fewer p16^Ink4a+^ cells were observed in lungs of ipilimumab-treated mice (Figure 7, A and B). The levels of p16^Ink4a^ cells decreased in the group treated with ipilimumab (Figure 7C).

We examined the ex vivo cytolytic effects of CD8^+^ T cells on senescent lung cells from mice injured by bleomycin (Figure 7D). Activation of these CD8^+^ T cells through ipilimumab-mediated CTLA4 blockade led to a reduction in β-GAL^+^ senescent cells from the bleomycin-injured lungs, as evidenced by decreased fluorescence signals, indicating enhanced senescent cell killing under conditions of CTLA4 blockade (Figure 7E). The direct cytotoxicity of ipilimumab-treated CD8^+^ T cells against sorted β-GAL^+^ senescent cells was further confirmed through live-cell imaging of coincubated cells (Figure 7, D–F; Supplemental Figure 2; and Supplemental Video 1). Collectively, these in vivo and ex vivo findings demonstrate the cytotoxic effects of CD8^+^ T cells on senescent cells and their improved killing efficiency when CTLA4 is inhibited.

## Discussion

Tissue-regenerative responses in mammals are highly orchestrated processes, involving intricate cell-cell interactions that either result in restoration of normal tissue architecture and function or in aberrant repair culminating in fibrosis (3). While fibrosis has traditionally been thought to represent the end stage of aberrant tissue repair or failed tissue regeneration, it is now increasingly recognized to be a dynamic and reversible process (2, 25); however, elucidation of mechanisms that resolve tissue fibrosis have lagged behind that of fibrosis development or initiation. Since most human fibrotic disorders that affect diverse organ systems come to clinical attention when they are either persistent or progressive, it is critical to understand biological mechanisms that not only retard progression of fibrosis but promote its resolution. In this report, we have identified a critical role for CD8^+^ cytotoxic T cells in immune surveillance and elimination of senescent cells that emerge during the lung injury repair process. Furthermore, we have identified the CTLA4 immune checkpoint protein as a critical contributor to persistent/progressive fibrosis in an animal model of repetitive genotoxic lung injury.

It is important to recognize that the actions of cytotoxic T cells are highly contextual and may have variable effects during the evolution of tissue repair and regeneration. Excessive activation of cytotoxic T cells might contribute to tissue injury, while eliminating infected, transformed, or senescent cells is predicted to be beneficial (26). In a previous study using immunodeficient NSG mice, CTLA4 blockade resulted in increased inflammation and fibrosis (27). This finding contrasts with our results and is likely explained by 3 major differences: (a) the use of immunocompetent versus severely immunodeficient mice; (b) fibrosis induced by repetitive bleomycin injury versus intravenous injection of IPF cells; and (c) initiation of CTLA4 blockade in the late phases of established fibrosis (5 weeks after the initial bleomycin dose and 3 weeks after a second dose) versus relatively early during fibrosis induction (7 days after intravenous injection of IPF fibroblasts). In humans with various cancers, the use of immune checkpoint blockade including CTLA4-targeted therapies has been associated with interstitial pneumonitis (28). Therefore, caution must be exercised when repurposing immune checkpoint blockade therapies in chronic lung inflammatory and fibrotic diseases, and further studies are required to determine if this approach will be beneficial in subgroups of patients with fibrotic lung disease.

While single-cell transcriptomics of isolated cells from normal and diseased tissues have the potential to identify novel cell populations and diverse differentiation states/fates, they lack spatial context. On the other hand, spatial transcriptomics/proteomics provide critical information regarding heterogenous cell populations within unique tissue niches; this allows for hypothesis-generating observations involving multiple cell types in diseased tissues (29). In our studies, we took an unbiased discovery approach to identify genes/proteins that were differentially expressed within IPF lung tissues, specifically within fibroblastic foci. The suggestion that cellular immune responses may be reduced in IPF lungs was unexpected, and this led to the discovery of upregulated expression of the immune checkpoint protein, CTLA4. Importantly, CTLA4 was not expressed on (myo)fibroblasts or epithelial cells within fibroblastic foci, but rather on CD8^+^ T cells within this fibrotic niche. In addition to confirming that CTLA4 is primarily expressed in T cell subpopulations, a search of larger single-cell RNA transcriptomic datasets of human IPF (IPF Cell Atlas; https://www.ipfcellatlas.com/) served to validate our original digital spatial observation that CTLA4 is upregulated in IPF lungs. Furthermore, immunofluorescence staining of IPF lungs confirmed the proximity of CTLA4-expressing CD8^+^ T cells and epithelial/fibroblastic cells within fibroblastic foci that are known to be enriched for senescent cells (4, 5).

These discovery-based studies of human IPF lung tissues led us to explore the functional role of CTLA4 in the context of lung injury repair using a mouse model of repetitive bleomycin injury that produces durable fibrotic responses (20). Importantly, the use of humanized CTLA4-knockin mice allowed us to interrogate the physiological consequences of targeting CTLA4 with a monoclonal antibody, ipilimumab, FDA-approved for various human cancers. As might be predicted, we observed an expansion of the Cd3*e*^+^ subpopulation of T cells with increased expression of *Icos* and *Tcf7* in lungs of ipilimumab-treated mice, supporting a derepressive effect of targeting CTLA4 in these cells. Remarkably, the activation of this cytotoxic T cell population was associated with a marked increase in the numbers of AT2 cells, facultative stem cells of the lung’s alveolar compartment (30). Both scRNA-Seq and immunofluorescence staining approaches confirmed the enhanced regenerative responses in association with diminished fibrosis in ipilimumab-treated mice. While the reciprocal antagonistic relationship between regenerative capacity of the alveolar epithelium and lung fibrosis is well appreciated (3, 31), our studies demonstrate a proregenerative effect of targeting CTLA4 and activating cytotoxic T cells during the fibrotic phase of lung injury repair. During this later reparative/remodeling phase and under the same conditions/context, we showed that CD8^+^ T cells isolated from bleomycin-injured mice, when treated with ipilimumab ex vivo compared to untreated CD8^+^ T cells, were more efficient in killing senescent cells exposed to bleomycin in vivo. Additionally, we showed that ex vivo derepression of CD8^+^ T cells with ipilimumab is capable of directly killing senescent fibroblasts. While there is biological plausibility to the concept that regenerative responses may be enhanced through the removal of senescent fibroblasts within regenerative niches (32), it is equally plausible that the elimination of senescent epithelial (progenitor) cells from these niches may also support the proliferative capacity of the nonsenescent progenitor subpopulation.

It is important to recognize that CTLA4 may be expressed on other cell types, in particular CD4^+^ Tregs, in which it may be constitutively expressed. The dependence on CTLA4 for the immunosuppressive function of Tregs is highly contextual, as CTLA4-independent immunosuppressive mechanisms have been identified (33). Furthermore, the roles of specific T cell subpopulations in the development and progression of fibrosis remains controversial, and their effects are likely to be stage specific (34). In our studies of established human IPF and persistent fibrosis following repetitive bleomycin injury in mice, we detected a higher induction/expression of CTLA4 in CD8^+^ (vs. CD4^+^) T cells, and activation of this sorted subpopulation was sufficient to induce cytotoxicity of senescent cells. However, we cannot exclude potential salutary effects of ipilimumab via its CTLA4 inhibitory action(s) on other immune cell (sub)populations.

Our studies are the first to our knowledge to show that activation of an endogenous T cell population can promote regeneration by targeting senescent cells that accumulate in fibrotic tissues. We have previously shown that senescent cells accumulate in IPF lungs (4), and others have shown that targeting these cells with a suicide gene-mediated ablation approach or with a senolytic cocktail of dasatinib plus quercetin mediates antifibrotic effects (5). Early-phase clinical studies have been initiated with the senolytic cocktail approach (24). Furthermore, CAR T cells engineered to target pathogenic, senescent cells have been shown to mediate antifibrotic effects (35, 36). However, the activation of *endogenous* immune-surveillance mechanisms to mediate proregenerative and antifibrotic effects has not been previously demonstrated.

Understanding of proregenerative roles of immune cells is critical in devising more effective therapies for chronic fibrotic disorders. Given the pervasive focus on the proinflammatory (and profibrotic) effects of immune cells, patients with IPF have traditionally been treated with potent immunosuppressive drug regimens. It was not until the first randomized, placebo-controlled clinical trial of an immunosuppressive drug cocktail in 2012, one that had become “standard of care” during the early 2000s, failed to show a beneficial effect that such approaches have been abandoned (37). Interestingly, patients treated with the immunosuppressive regimen in this clinical trial demonstrated (unexpectedly) worse outcomes with higher mortality and increased hospitalization rates compared to individuals in the control group, suggesting that immune pathways may be protective in patients with IPF with established fibrosis. Although this requires further investigation, it is plausible that, based on the results of the findings reported here, steroids and other immunosuppressive agents may confer unanticipated detrimental, profibrotic effects through loss of immune-surveillance functions. On the contrary, activating these endogenous immune mechanisms may promote proregenerative tissue repair responses.

## Methods

### Sex as a biological variable.

For human tissue-based studies, both male and female subjects were included. For mouse studies, only male mice were studied due to the higher incidence and prevalence of IPF in males.

### Human lung tissue specimen.

Biospecimens of patients with IPF and individuals acting as controls were obtained from a biorepository established through an NIH Program Project Grant (P01 HL114470).

### Spatial transcript profiling — nanoString GeoMx.

For the nanoString GeoMx Transcriptomics Assay, tissue slides were prepared according to the protocol provided in the user manual (NanoString, MAN-10100-02). In brief, 5 μm sections of FFPE lung tissue from patients with IPF and healthy individuals acting as controls were examined for spatial proteomics. The Human Whole Transcriptome Atlas, comprising 18,676 RNAs, was utilized on the GeoMx DSP Platform (NanoString Technologies) to capture genetic data from transcribed mRNA. Morphological markers, including pan-CK (a marker for lung epithelium), CD31 (a marker for vascular endothelium), and α-SMA (a marker for smooth muscle cells and myofibroblasts), were employed to visualize ROIs. For IPF samples, ROIs were selected to capture fibroblastic foci enriched in α-SMA within alveolar regions. In healthy control lung tissues, ROIs were randomly chosen to represent normal-appearing areas of airway epithelium, blood vessels, and alveoli. After incubation (mRNA probe) and hybridization (morphological markers), the slides were loaded into the GeoMx DSP instrument, which utilizes a programmable digital micromirror device to direct UV light at specific ROIs. This light activation releases photolabile indexing oligonucleotides in a region-specific manner. The released oligos were collected through microcapillary aspiration, dispensed into a microtiter plate, and quantified using next-generation sequencing for digital counting. A total of 18,676 genes were assayed using the NanoString Human Whole Transcriptome Atlas. The DSP instrument was used to capture high-resolution wide-field scans of each tissue section, from which ROIs were selected for UV-induced oligo collection. The barcoded oligos were then collected and digitally quantified using an Illumina sequencer.

### Spatial protein profiling — nanoString GeoMx.

For the nanoString GeoMx proteomics assay, slides were prepared according to the user manual (nanoString, MAN-10100-01) as previously outlined. This study utilized antibodies targeting 79 proteins (Supplemental Table 1) from 7 modules: human immune cell profiling, immune activation status, immune cell typing, PI3K/AKT signaling, pan-tumor, nCounter cell death, and MAPK signaling, along with internal control housekeeping proteins. A cocktail of these primary antibodies was conjugated to photocleavable oligonucleotides. Additionally, morphological marker antibodies for pan-CK, CD31, and α-SMA were included in the mix of oligonucleotide-conjugated antibodies to visualize ROIs. All 24 ROIs were selected using the nanoString GeoMx DSP System (33). Oligos from the selected ROIs were released through UV exposure. The resulting digital counts were mapped back to each ROI, generating a detailed map of protein. These digital counts were normalized using a signal-to-noise ratio and internal control housekeeping proteins (histone H3, S6, GAPDH). Data analysis and heatmap generation were carried out using the GeoMx DSP system software (nanoString Technologies).

### Immunofluorescence staining.

Consecutive sections from control and IPF lung tissues were processed for immunofluorescence staining. These unstained sections were incubated at 65°C and were then allowed to stand at room temperature for 45 minutes. The remaining paraffin was removed through immersion in xylene. The tissue sections were gradually hydrated by sequential immersion in a graded alcohol series (absolute ethanol, 98%, 95%, 70% ethanol) and, finally, in water. Antigens were unmasked by steaming in a coplin jar filled with Diva decloaker antigen retrieval solution (Biocare, V2004). The slides were cooled for 20 minutes and washed with deionized water. Nonspecific antibody binding was blocked using a background blocking solution (Biocare, BS966). Lung sections were incubated with primary antibodies against CTLA4 (Biocare, API3211AA), CD8 (Biocare, ACI3160AA), CD4 (Biocare, API3209AA), and α-SMA (Cell Signaling Technology,19245). Primary antibodies were visualized with donkey anti-rabbit secondary antibody, Alexa Fluor 594 (Thermo Fisher Scientific, A32754), and goat anti-mouse Alexa Fluor 488 (Thermo Fisher Scientific, A32723). Sections were washed (PBS/Tween 0.01%) and mounted with ProLong Gold antifade mount with DAPI (Thermo Fisher Scientific, P36941). Images were captured using a fluorescence microscope (Keyence, BZ-X716) and representative images are presented.

### Spatial transcript profiling — Xenium.

Xenium In Situ Platform was utilized for subcellular mapping of 289 genes (Xenium V1) in lungs of 3 individuals with IPF. Each probe in this system consists of 2 paired sequences that are complementary to the targeted mRNA, along with a unique gene-specific barcode. When these paired sequences bind to their target, they undergo ligation, forming a circular probe. This circular structure is then amplified through rolling circle amplification, which enhances the signal-to-noise ratio and improves target detection and decoding.

### Sample processing.

Tissue samples were submitted to the COBRE Multiomics Core at Tulane University for processing. In brief, sample preparation involved rehydrating and sectioning FFPE blocks with a microtome (Leica, RM2135) to obtain 5 μm sections, which were then mounted onto Xenium slides (10x Genomics). After an overnight drying period, the slides were stored in a sealed desiccator at room temperature for up to 7 days before being placed in imaging cassettes for further processing. The deparaffinization and decrosslinking steps rendered subcellular RNA targets accessible. Gene panel probe hybridization was conducted overnight at 50°C using a Bio-Rad DNA Engine Tetrad 2. Following this, unbound probes were washed away. Ligase was then introduced to circularize the paired ends of the bound probes at 37°C for 2 hours, followed by rolling circle amplification at 30°C for 2 hours. The prepared slides were stored in PBS-T in the dark at 4°C for up to 5 days before being analyzed with the Xenium Analyzer (please refer to 10x Genomics protocols CG000578 and CG000580).

### Post-Xenium H&E.

After the Xenium run, slides were removed from the Xenium Analyzer instrument, and the quencher was removed according to 10x Genomics’ Demonstrated Protocol CG000613. Immediately following, the slides were stained with H&E. H&E-stained sections were scanned using the Vectra Polaris Automated Quantitative Pathology Imaging System (Akoya Biosciences).

### Animal studies.

hCTLA4 mice were purchased from genOway and housed at the Tulane University Vivarium. We used 12-week-old male mice for this study. hCTLA4 mice were anesthetized with isoflurane/oxygen and were administered bleomycin via the oropharyngeal route. Each mouse was administered 1.25 U per kg body weight bleomycin in 2 doses, 2 weeks apart. On day 21, the control bleomycin group received IgG1 isotype control (Bio X cell, BE0297), and the treatment bleomycin group received ipilimumab (Bio X cell, SIM0004) at a concentration of 5 mg per kg body weight twice a week for 4.5 weeks by intraperitoneal injection. At the end of the treatments, the mice were euthanized; the right lungs were collected for single-cell RNA-Seq, and the left lungs were collected for histologic analysis.

### Single-cell RNA-Seq.

A million cells were collected from the right lung of mice treated with either IgG1 isotype control or ipilimumab. The lungs were minced into ~1 mm^3^ pieces using a scalpel prior to enzymatic dissociation. The minced sections were then transferred into a 50 mL conical tube containing 10 mL enzymatic digestion mix (collagenase 1 mg/mL with DNase I in 9 mL serum-free DMEM). The conical tubes were then placed on a 37°C rocking shaker (at 220 rpm) for 30 minutes. The digestion mixture was then filtered using a 70 μm cell strainer. Cells were then washed 10% of complete DMEM (500*g* for 5 minutes at 4°C). The resulting pellet was resuspended in 1 mL PBS (containing 0.04% BSA). Using a Cellometer, we validated cell numbers and viability counts before the preparation of the scRNA. A commercially available 10x single cell RNA seq technology kit (10x Genomics) was used to evaluate 5,000 live cells from each sample. Single-cell suspensions were partitioned into nanoliter-scale Gel beads-In-Emulsion. Full-length barcoded cDNAs were then generated and amplified by PCR to obtain sufficient mass for library construction. Single-cell 3′ libraries with standard Illumina P5 and P7 paired-end constructs were prepared by enzymatic fragmentation, end-repair, polyA-Tailing, and adaptor ligation. Library quality control tests were performed by using an Agilent High Sensitivity DNA kit with Agilent 2100 Bioanalyzer and quantified using a Qubit 2.0 fluorometer. Libraries were pooled to a final concentration of 1.8 pM and were sequenced with paired-end single index configuration using Illumina NextSeq 2000. Cell Ranger version 6.1.2 (10x Genomics) was used to aligned scRNA-Seq data to mm10 mouse. Soup X v1.6.2 was used to analyze 10x Cell Ranger output with default parameters to remove contaminating ambient RNA. The ambient RNA-corrected Soup X gene matrix output files were then analyzed using Seurat suite (version 4.2.0) (38), and low-quality cells were further excluded from the downstream analysis. For clustering, principal-component analysis was performed for dimension reduction. The top 10 principal components were selected using a permutation-based test implemented in Seurat and analyzed with uniform manifold approximation and projection (UMAP) for clustering visualization. Differentially expressed genes were identified for each cluster with the “Find All Markers” function, and cell types were assigned using the cell markers.

### Lung hydroxyproline measurements.

A colorimetric assay kit from Sigma-Aldrich (catalog MAK008) was used to measure hydroxyproline content in whole lungs of experimental mice. The reaction of oxidized hydroxyproline with 4-dimethyl amino benzaldehyde yielded a calorimetric product proportionate to the amount of hydroxyproline present, which was used to calculate the hydroxyproline concentration using the standards provided. Whole lungs from mice were weighed and homogenized in water (10 mg tissue/100 μL H_2_O). The homogenized samples were then hydrolyzed by incubating them for 3 hours at 120°C with 12N hydrochloric acid. The hydrolyzed sample was mixed and centrifuged at 10,000*g* for 3 minutes, and 10 mL clear supernatant was transferred to a 96-well plate and placed in a 60°C oven to dry samples. Chloramine T/Oxidation Buffer Mixture Diluted DMAB Reagent (Sigma-Aldrich, catalog MAK008) was freshly prepared according to the manufacturer’s instructions. After the addition of 100 μL Chloramine T/Oxidation Buffer (5-minute incubation at room temperature) and 100 μL diluted AMAB reagent to each well, the plate was incubated for 90 minutes at 60°C, and absorbance was measured at 560 nm.

### Mouse histological analysis.

At the end of the animal experiments, mice were euthanized, and the lungs were perfused with PBS. After perfusion, the left lobe of the mouse lung was fixed in 10% neutral buffered (pH 7.4) formalin for 24 hours at room temperature. The lung tissues were paraffin-embedded and sectioned at 5 μm thickness. These sections were utilized for H&E staining and Masson’s trichrome staining as described previously (4). Slides were scanned on Vectra Polaris Automated Quantitative Pathology Imaging System (Akoya Biosciences). Trichrome-stained areas were quantified with machine-learning algorithms using inForm automated image analysis software (Akoya Biosciences). H&E staining was quantified using QuPath software.

### Multispectral fluorescence immunohistochemistry pro-SPC.

Multispectral fluorescence immunohistochemistry staining was performed using an Opal 7-Color Automation IHC Kit (Akoya Biosciences), following the manufacturer’s guidelines. The following primary antibodies were used: pro-SPC (Abcam, ab90716, 1:500), α-SMA (Cell Signaling Technology, 19245, 1:100), CD8 (Cell Signaling Technology, 98941, 1:100), and CTLA4 (Cell Signaling Technology, 53560, 1:100). The protocol involved deparaffinizing the slides, performing antigen retrieval, and blocking with antibody diluent (provided in the kit). The slides were incubated overnight with the primary antibodies, followed by a 10-minute incubation with HRP-conjugated secondary polymer (provided in the kit). Slides were then incubated with HRP-reactive OPAL fluorescent reagents Opal 480 (CD8), Opal 520 (for α-SMA), Opal 570 (for pro-SPC), and Opal 690 (CTLA4); staining with each OPAL was performed as instructed by the manufacturer (Akoya Biosciences). After incubation, the sections were counterstained with DAPI and mounted. Imaging was performed using the Vectra Polaris Automated Quantitative Pathology Imaging System (Akoya Biosciences). Spectrally unmixed images were generated using Akoya Biosciences v1.4.8 software, and further analysis was conducted using machine-learning algorithms in the inForm software. Tissue regions were categorized into pro-SPC^+^ and “other” (cells negative for pro-SPC). The cell phenotype classification, implemented in the inForm software, was based on multinomial logistic regression using image features derived from texture analysis and cell segmentation. The density of pro-SPC^+^ cells (cells/mm²) was calculated.

### Multispectral fluorescence immunohistochemistry p16^INK4A^.

Multispectral fluorescence immunohistochemistry staining was performed as described above. The Opal 3-Plex Anti-Rb Manual Detection Kit (Akoya Biosciences, NEL840001KT) was used for multiplexing. The following antibodies were used: pro-SPC (abcam, ab90716, 1:500) and p16^INK4A^ (Abcam, EP1551Y, 1:100). The protocol involved deparaffinizing the slides, performing antigen retrieval, and blocking with the antibody diluent (provided in the kit). The slides were then incubated with the primary antibodies, followed by incubation with the HRP-conjugated secondary polymer (provided in the kit). Subsequently, the slides were incubated with HRP-reactive OPAL fluorescent reagents: Opal 520 (pro-SPC) and Opal 690 (p16^INK4A^). Images of the tissue regions were captured using the inForm software package, and p16INK4A expression was analyzed.

### Flow cytometry.

Lungs were minced into ~1 mm^3^ pieces using a scalpel prior to enzymatic dissociation. The minced sections were then transferred into a 50 mL conical tube containing 10 mL enzymatic digestion mix (1 mg/mL collagenase with DNase I in 9 mL serum-free DMEM). The conical tubes were then placed on a 37°C rocking shaker (220 rpm) for 30 minutes. The digestion mixture was then filtered using a 70 μm cell strainer. Cells were then washed with 10% complete DMEM (500*g* for 5 minutes at 4°C). The resulting pellet was resuspended in 0.2 mL PBS. Cells were then stained for flow cytometry. The antibodies used included anti-CD45 (BioLegend, 103115), CD3 (BioLegend, 100205), CD8 (BioLegend, 140403), CD4 (BioLegend, 100506), CD152 (BioLegend, 130-116-931), pro-SPC (Santa Cruz Biotechnology, sc-518029), EpCAM (Thermo Fisher Scientific, 17-5791-80), and Ki67 (Santa Cruz Biotechnology, sc-23900).

### T cell–mediated killing assay.

hCTLA4 mice were anesthetized with isoflurane/oxygen and exposed to 2 doses of bleomycin (1.25 U per kg body weight, oropharyngeal route) administered 2 weeks apart. Five weeks after the first bleomycin dose, mice were euthanized, and lungs were harvested for cell isolation. In brief, lungs were minced into approximately 1 mm^3^ pieces using a scalpel prior to enzymatic dissociation. The minced sections were then transferred into a 50 mL conical tube containing 10 mL enzymatic digestion mix (collagenase, 1 mg/mL with DNase I in 9 mL of serum-free DMEM). The conical tubes were then placed on a 37°C rocking shaker (220 rpm) for 30 minutes. The digestion mixture was then filtered using a 70 μm cell strainer. Cells were then washed with 10% DMEM and centrifuged (500*g* for 5 minutes at 4°C). The resulting pellet was resuspended in 1 mL PBS containing 0.04% BSA; the major fraction of these cells (70%) was used for CD8^+^ T cell purification using negative selection with a commercial kit (STEMCELL Technologies, catalog 19853). The remaining 30% of (whole lung) cells were stained with SPiDER-β-GAL (DoJindo, SG04-1) and plated into 48-well plate. Next, these cells cocultured with purified CD8^+^ T cells at a 2:1 ratio (0.3 × 10^4^ nonpurified cells and 0.15 × 10^4^ CD8^+^ T cells) in the presence and absence of ipilimumab (SIM0004, Bio X cell; 0.05 μg/mL) for 12 hours. The CD8^+^ T cell–mediated killing with resulting loss of green signals was assessed by the IncuCyte system (IncuCyte SX5 Live-Cell Analysis System (Sartorius). In parallel, we designed an assay to directly visualize killing of senescent fibroblasts by CD8^+^ T cells in the presence and absence of ipilimumab. Fibroblasts were isolated from bleomycin-injured lungs by adherence purification as described above. After 2 weeks, adherent fibroblasts were stained with SPiDER-β-GAL (DoJindo, SG04-1) and flow sorted to isolate senescence-associated βGal–expressing fibroblasts. CD8^+^ T cells were stained with red (Biotium, 30089) dye for 15 minutes and washed with PBS to remove unbound stain. The isolated senescence-associated βGal–expressing fibroblasts were cocultured with labeled CD8^+^ T cells iatn a 2:1 ratio with and without ipilimumab treatment at the concentration of 0.05 μg/mL for 12 hours (as described above for nonpurified cells). Images of the cocultured cells were acquired with the IncuCyte SX5 Live-Cell Analysis System (Sartorius).

### Statistics.

Statistical comparisons between groups were performed using the Mann-Whitney test or 2-tailed Student’s *t* test. Statistical analysis was performed with GraphPad Prism 10.0.2. *P* values of less than 0.05 were considered significant.

### Study approval.

The institutional review board at Tulane University, New Orleans, Louisiana, USA, approved the use of deidentified patient specimens. Mouse studies were conducted in accordance with protocols approved by the Tulane University Institutional Animal Care and Use Committee (protocol no. 1196).

### Data availability.

Data related to the paper are available from the corresponding author on reasonable request. scRNA-Seq data were deposited in the Gene Expression Omnibus (GEO) database (GSM7657238), release date May 15, 2025.

## Author contributions

SY and VJT conceived the project, designed experiments, analyzed the data, and wrote the manuscript. SY, MA, DP, YM, and JM conducted or assisted with experiments. SY and SK performed bioinformatics analyses. JBM, JAL, and JK provided intellectual input and assisted with manuscript writing.

## Supplementary Material

Supplemental data

Supplemental table 1

Supplemental video 1

Supporting data values

## Version 1 03/18/2025

In-Press Preview

## Version 2 05/15/2025

Electronic publication
