# Supplementary material for: Reactivation of CTLA4-expressing T cells accelerates resolution of lung fibrosis in a humanized mouse model
Source: J Clin Invest. 2025 Mar 18;135(10):e181775. doi: 10.1172/JCI181775 (PMC12077895; doi:10.1172/JCI181775)
Supplement: Supplemental data [file jci-135-181775-s243.pdf]

SUPPLEMENTARY FIGURES 1

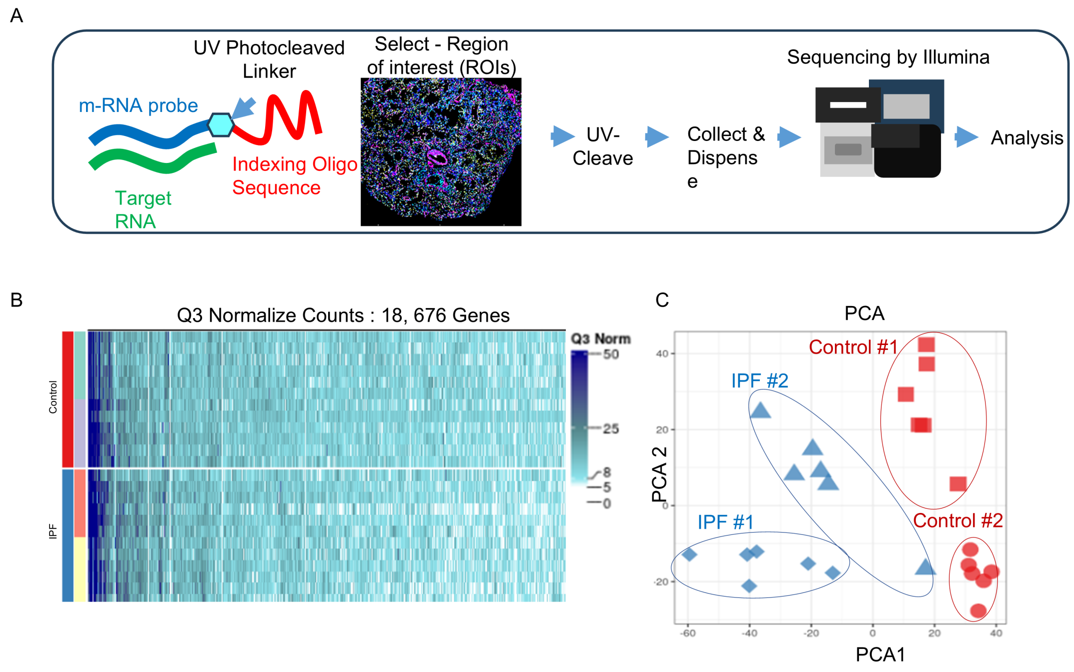

SUPPLEMENTARY FIGURES 2

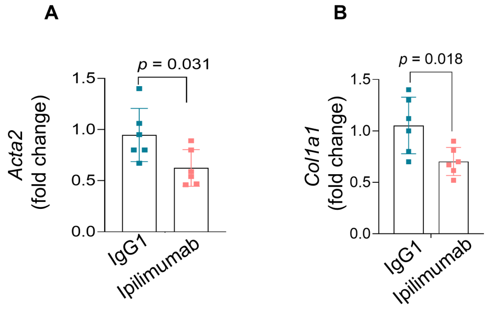

SUPPLEMENTARY FIGURES 3

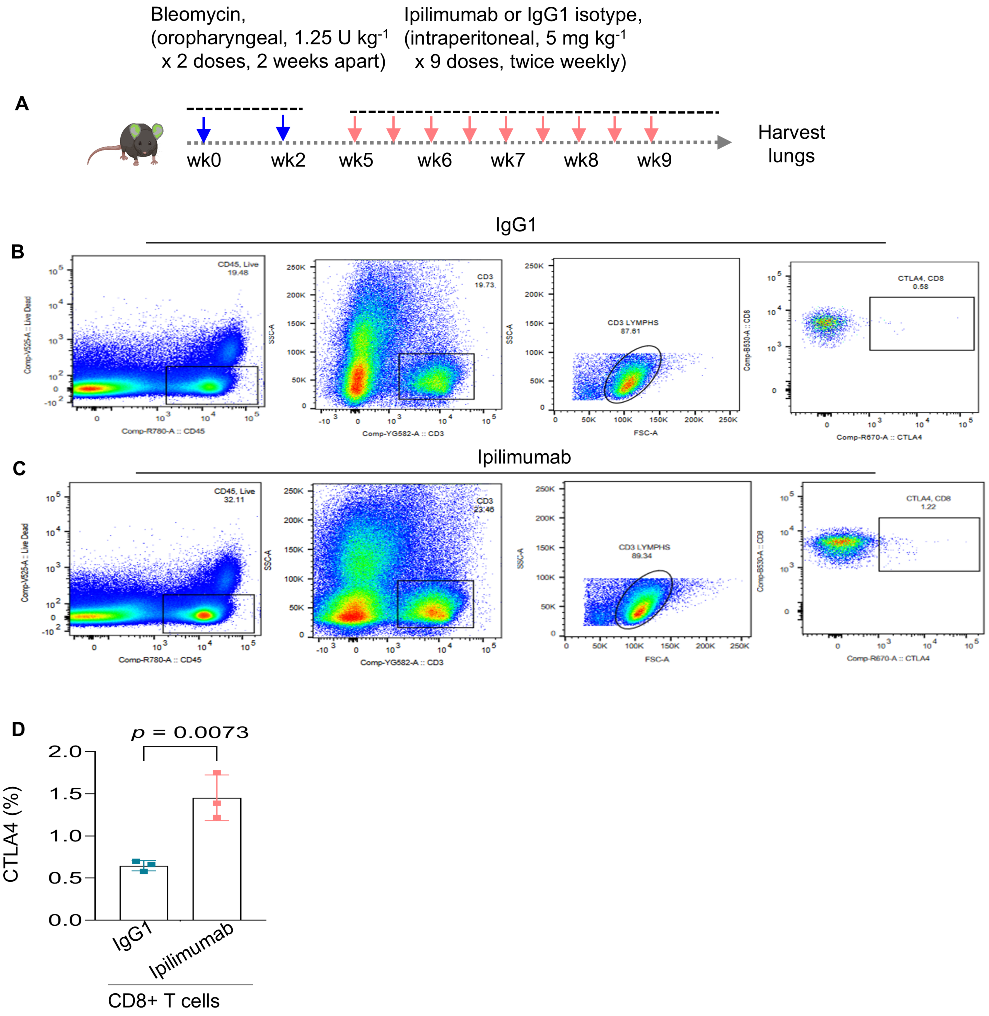

SUPPLEMENTARY FIGURES 4

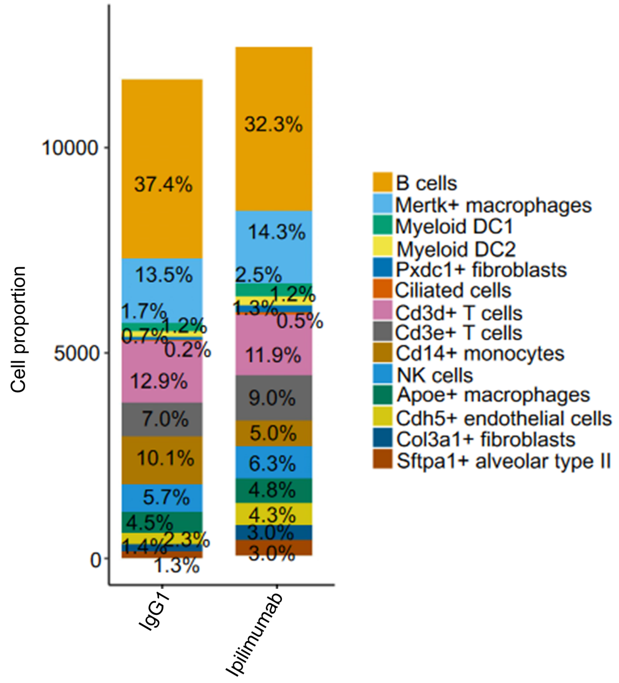

SUPPLEMENTARY FIGURES 5

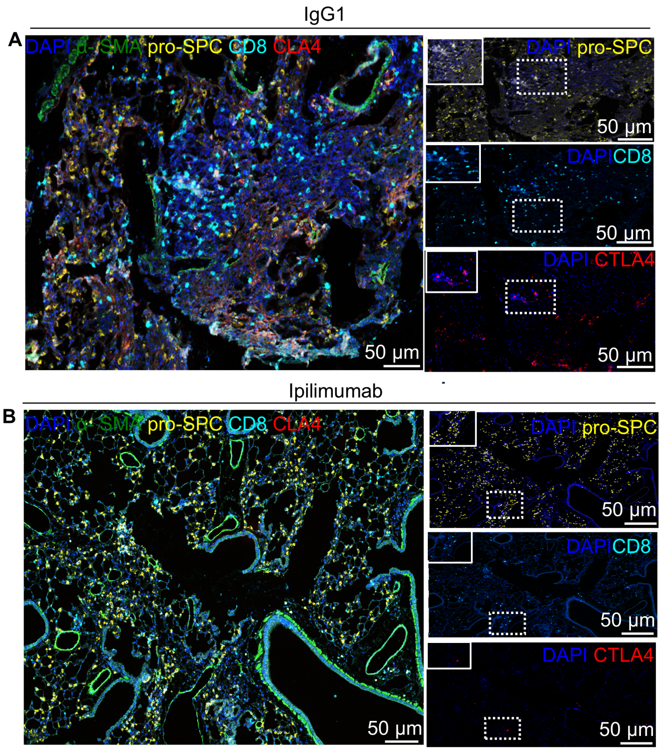

Supplemental Figure Legends

Supplemental Figure 1: Schematic of study design and workflow. (A) Tissue sections were labeled with in-situ probes for mRNA detection via a UV photocleavable linker and 4 fluorescently labeled morphology markers; pan-CK (cyan), CD31(yellow), α-SMA (magenta), and the DAPI (blue). (B) Heatmap displaying normalized read counts for control and IPF samples. (C) PCA plot depicting the distribution of control (n = 12 ROIs) and IPF samples (n = 12 ROIs).

Supplemental Figure 2: Gene expression of Acta2 and Col1a1 in lungs of mice treated with IgG1 control or ipilimumab (n = 6 per group). Data are reported as mean ± SEM; statistical differences were tested using a paired Student's t-test (two-tailed).

Supplemental Figure 3: Analysis of CTLA4-expressing CD8+ T cells in lungs of IgG1 and Ipilimumab treated groups. Data are reported as mean ± SEM; statistical differences were tested using a paired Student's t-test (two-tailed). (A) Experimental outline of humanized CTLA4 mice subjected to repetitive bleomycin injury induced pulmonary fibrosis via oropharyngeal administration of bleomycin (1.25 U kg-1 body weight), two doses,14 days apart. On day 21 after the 2nd dose, mice were treated intraperitoneally with either control IgG1 isotype or the anti-CTLA4 monoclonal antibody ipilimumab at doses of 5 mg kg-1 body weight twice per week for 4.5 weeks (total of 9 doses). (B-C) Representative flow cytometry plots of lung cells isolated from IgG1 and ipilimumab treated groups and stained for CD45, CD3, CTLA, and CD8. (D) Bar graph depicting percentage of CTLA4 expressing CD8+ T cells from IgG1 isotype control and ipilimumab treated groups from B-C. Data are reported as mean ± SEM; statistical differences were tested using a paired Student's t-test (two-tailed).

Supplemental Figure 4: Cell type annotation and relative proportions from scRNA-seq of IgG1 and ipilimumab treated groups.

Supplemental Figure 5: Multiplex IHC staining of lungs from IgG1 control and ipilimumab treated mice. (A) Representative multiplex IHC staining with a-SMA (green), pro-SPC (yellow), CD8 (cyan), and CTLA4 (red) in IgG1 group.(B) Representative multiplex IHC staining with a-SMA (green), pro-SPC (yellow), CD8 (cyan), and CTLA4 (red) in the ipilimumab treated group.

Supplemental Table 1: Ingenuity pathway analysis (IPA) of differentially expressed genes between IPF and control lungs.

Supplemental Table 2: Differences in protein expression within regions of interest (ROIs) in control vs. IPF lungs (n = 12 ROIs in each group).

Supplemental Table 3: Differentially expressed genes in the AT2 cell cluster, comparing ipilimumab and control IgG1 treated mice (n = 3 per group).

Supplemental Video 1: Representative video of CD8+ T cell mediated killing of SPiDER-β-GAL positive lung cells from bleomycin injured mice (based on protocol depicted in Figure 4D). CD8+ T cells and SPiDER-β-GAL+ cells were isolated from bleomycin-injured mice and co-cultured in a 1:2 ratio for 14 h with or without ipilimumab.

<u>Supplemental Table 3</u>: Differentially expressed genes in the AT2 cell cluster, comparing ipilimumab vs. control groups

Gene	p value	avg_log2FC	Ipilimumab	IgG1	p_value adjusted
Cd74	4.41E-20	2.584728	0.601	0.21	9.68E-16
Sftpa1	6.69E-14	2.512903	0.412	0.09	1.47E-09
Lyz2	1.25E-10	2.458137	0.518	0.287	2.74E-06
Sftpc	8.31E-14	2.345374	0.404	0.078	1.82E-09
Cxcl15	1.49E-11	2.154575	0.365	0.09	3.28E-07
Sftpb	4.82E-13	2.02476	0.404	0.09	1.06E-08
Sftpd	8.82E-11	1.976848	0.368	0.102	1.94E-06
Igkc	8.96E-09	1.949	0.334	0.114	0.000197
Chil1	2.77E-08	1.831805	0.275	0.066	0.000608
Scd1	3.14E-10	1.799241	0.383	0.126	6.89E-06
Slc34a2	2.58E-11	1.730396	0.332	0.054	5.66E-07
H2-Aa	5.59E-10	1.703045	0.386	0.138	1.23E-05
Napsa	4.36E-08	1.568181	0.329	0.12	0.000957
Rps24	7.13E-18	1.54677	0.847	0.677	1.57E-13
Npc2	1.45E-08	1.531377	0.648	0.515	0.000318
H2-Eb1	1.17E-07	1.477867	0.342	0.144	0.00257
H2-Ab1	4.56E-09	1.445593	0.417	0.18	0.0001
Ank3	4.28E-09	1.357641	0.355	0.114	9.41E-05
Btg1	1.77E-06	1.32052	0.51	0.377	0.038929
Wfdc2	4.85E-08	1.309648	0.376	0.15	0.001065
Hc	5.56E-07	1.266914	0.215	0.042	0.012204
Rps15a	5.01E-11	1.264492	0.785	0.683	1.10E-06
S100g	3.74E-07	1.233636	0.244	0.06	0.008207
Rpl32	2.00E-13	1.215655	0.759	0.575	4.39E-09
Rps7	1.28E-09	1.196044	0.744	0.653	2.81E-05
Rps19	1.15E-10	1.183598	0.793	0.677	2.53E-06
Rpl30	1.10E-12	1.178808	0.788	0.635	2.41E-08
Rps18	6.70E-11	1.168032	0.731	0.581	1.47E-06
Rps10	3.22E-10	1.156141	0.793	0.713	7.06E-06
Lamp3	4.71E-07	1.149806	0.254	0.066	0.010333
Fau	1.56E-13	1.142465	0.868	0.737	3.42E-09
Rpl21	6.05E-12	1.141484	0.79	0.695	1.33E-07
Rpl3	3.33E-07	1.138114	0.684	0.611	0.007304
Rps27	2.95E-10	1.115258	0.811	0.731	6.48E-06
Rpl23	5.44E-13	1.112567	0.832	0.725	1.20E-08
Rpl13a	4.97E-10	1.1122	0.767	0.665	1.09E-05
Rps26	7.70E-08	1.110515	0.744	0.629	0.001692
Rps28	3.94E-09	1.096245	0.699	0.545	8.65E-05

Rps23	1.48E-10	1.090065	0.79	0.695	3.24E-06
Rps20	6.30E-09	1.077824	0.785	0.737	0.000138
Rps16	1.29E-10	1.066553	0.782	0.659	2.83E-06
Rps3	2.46E-09	1.055745	0.712	0.611	5.39E-05
Rps14	3.74E-11	1.048917	0.821	0.749	8.22E-07
Rpl10a	2.92E-07	1.044571	0.65	0.545	0.006403
Rps3a1	8.07E-10	1.028929	0.79	0.701	1.77E-05
Rpl12	1.17E-07	1.012892	0.674	0.533	0.002577
Rpl36	1.85E-06	0.998969	0.687	0.611	0.040657
Rpl26	8.62E-10	0.993438	0.788	0.671	1.89E-05
Rpl18a	9.30E-09	0.98233	0.793	0.76	0.000204
Rps4x	2.18E-08	0.969563	0.736	0.653	0.000478
Rpl35a	1.32E-08	0.968654	0.788	0.719	0.00029
Rps21	1.87E-09	0.966887	0.785	0.725	4.10E-05
Cbr2	1.13E-07	0.965579	0.311	0.09	0.00248
Rps12	6.62E-11	0.956274	0.816	0.725	1.45E-06
Rpl37a	1.47E-07	0.956256	0.793	0.76	0.003225
Tpt1	7.67E-17	0.944778	0.948	0.862	1.68E-12
Rpl8	2.61E-08	0.93508	0.788	0.695	0.000573
Rps29	3.01E-09	0.924339	0.816	0.725	6.61E-05
Rpl9	3.68E-09	0.923491	0.756	0.713	8.09E-05
Rpl28	1.04E-08	0.903318	0.746	0.683	0.000228
Rpl17	1.77E-07	0.890904	0.777	0.737	0.003878
Rpl18	1.13E-07	0.888374	0.746	0.695	0.002484
Rpl34	1.58E-08	0.883137	0.795	0.725	0.000346
Rpl39	8.34E-07	0.874634	0.715	0.605	0.018308
Rpl13	2.41E-09	0.861614	0.832	0.772	5.29E-05
Rps11	2.48E-07	0.854399	0.767	0.707	0.005442
Tmsb4x	4.76E-14	0.853507	0.938	0.88	1.05E-09
Rps5	1.17E-07	0.847956	0.764	0.671	0.00256
Rpl19	1.69E-09	0.844439	0.826	0.725	3.70E-05
Rplp1	1.09E-08	0.843199	0.847	0.772	0.00024
Rpl27a	4.01E-09	0.825784	0.832	0.766	8.82E-05
Fth1	1.23E-10	0.802682	0.868	0.808	2.70E-06
Rpl37	1.79E-08	0.801493	0.767	0.683	0.000393
Eef1a1	4.19E-12	0.77788	0.922	0.886	9.20E-08
Rps8	8.54E-07	0.740132	0.819	0.766	0.018751
Rpl41	6.04E-08	0.626984	0.847	0.826	0.001327
Actb	1.09E-08	0.602382	0.977	0.97	0.00024
Ftl1	9.50E-07	0.522161	0.813	0.784	0.020859
Tbc1d8	3.49E-07	-0.25335	0.246	0.467	0.007661

Dmxl2	6.80E-07	-0.26041	0.101	0.263	0.014938
Map3k7	5.10E-08	-0.27378	0.153	0.359	0.00112
Mfsd1	5.62E-07	-0.27571	0.166	0.353	0.01234
Adam10	1.68E-06	-0.27693	0.386	0.599	0.036885
Fam192a	1.70E-06	-0.27922	0.14	0.305	0.037299
Kidins220	1.84E-07	-0.27949	0.29	0.527	0.004051
Cab39	7.08E-07	-0.28306	0.358	0.605	0.015545
Zfp532	5.65E-07	-0.28756	0.228	0.443	0.012405
Rassf8	1.20E-06	-0.28893	0.21	0.407	0.026324
Rab7	1.88E-06	-0.306	0.456	0.754	0.041209
Pcdh17	2.23E-06	-0.30976	0.383	0.647	0.048946
Layn	1.19E-06	-0.3103	0.039	0.156	0.026183
Mob1a	1.98E-08	-0.32314	0.264	0.509	0.000435
Apbb2	1.86E-06	-0.32734	0.604	0.856	0.04078
Gcnt2	1.06E-06	-0.32769	0.394	0.653	0.023198
Irf9	2.25E-06	-0.3294	0.168	0.353	0.049384
Atxn7l3b	6.05E-07	-0.3333	0.358	0.587	0.013294
Rasip1	1.68E-06	-0.34222	0.378	0.653	0.036942
Ehd2	1.76E-08	-0.34546	0.319	0.611	0.000387
Pdgfb	3.06E-07	-0.34563	0.446	0.707	0.006709
Orc5	1.14E-09	-0.3488	0.028	0.174	2.51E-05
Crk	1.66E-07	-0.34881	0.396	0.653	0.003636
Git2	7.34E-09	-0.35137	0.337	0.611	0.000161
Psmd12	2.09E-07	-0.35146	0.262	0.479	0.004588
Clstn1	2.80E-07	-0.353	0.433	0.677	0.006155
Golim4	2.36E-07	-0.35409	0.171	0.365	0.005179
Sppl2a	1.22E-07	-0.35492	0.402	0.659	0.002684
Cds2	1.34E-08	-0.35545	0.368	0.641	0.000294
Mylk	9.27E-07	-0.3609	0.448	0.707	0.020362
Ptpra	5.56E-07	-0.36814	0.306	0.539	0.012209
Pon2	5.13E-07	-0.36905	0.373	0.611	0.011271
Zfp710	8.09E-09	-0.36914	0.373	0.647	0.000178
A4galt	2.29E-07	-0.36958	0.161	0.359	0.00502
Nid1	1.61E-06	-0.37005	0.269	0.485	0.03529
Gm9917	2.42E-07	-0.37059	0.218	0.443	0.00532
Elovl5	7.52E-07	-0.37377	0.391	0.629	0.016512
Efnb1	6.49E-07	-0.37617	0.342	0.581	0.014255
Sbf2	1.22E-07	-0.37943	0.404	0.677	0.002689
Dhrs3	2.91E-07	-0.38985	0.409	0.647	0.006394
Sh2b3	2.02E-06	-0.3905	0.207	0.413	0.044324
Togaram1	9.30E-07	-0.39194	0.212	0.413	0.02043

Kif13a	8.31E-07	-0.3929	0.386	0.617	0.018259
Soga1	2.67E-09	-0.3933	0.176	0.419	5.86E-05
Ripply3	8.92E-07	-0.39601	0.308	0.539	0.019581
Plscr4	8.81E-07	-0.39689	0.259	0.473	0.019356
Pawr	1.80E-06	-0.39835	0.365	0.593	0.039432
Atrn	2.24E-07	-0.40178	0.254	0.479	0.004928
Wars	7.62E-07	-0.40357	0.352	0.581	0.016734
Jcad	1.45E-06	-0.40518	0.254	0.467	0.031818
Ppp1r13b	9.98E-07	-0.40678	0.402	0.617	0.021907
Mrtfb	1.09E-06	-0.40724	0.311	0.521	0.023954
Rock2	1.97E-07	-0.40756	0.619	0.844	0.004317
Pacs1	2.26E-06	-0.40778	0.43	0.641	0.049647
Tcf4	2.24E-06	-0.40971	0.635	0.856	0.049114
Notch1	1.20E-07	-0.41068	0.383	0.635	0.002632
Stip1	1.50E-06	-0.41299	0.145	0.305	0.032832
Lars2	1.64E-06	-0.41353	0.699	0.886	0.036037
Col4a1	5.27E-07	-0.41427	0.536	0.802	0.011562
Capn2	3.32E-10	-0.4147	0.324	0.617	7.29E-06
Tanc1	1.19E-07	-0.4152	0.554	0.814	0.002619
St3gal6	4.04E-07	-0.41577	0.435	0.683	0.00888
Vat1	6.70E-07	-0.41645	0.409	0.659	0.014721
Rab31	9.98E-07	-0.41651	0.342	0.593	0.021915
N4bp1	6.93E-08	-0.41692	0.238	0.467	0.001523
Plxna1	1.74E-08	-0.41845	0.342	0.581	0.000382
Ushbp1	5.60E-08	-0.41928	0.192	0.407	0.001229
Tspan12	1.43E-08	-0.41928	0.464	0.719	0.000315
Pde8b	7.83E-07	-0.4196	0.472	0.713	0.017199
Ppp2r5a	1.56E-06	-0.42008	0.57	0.802	0.034359
Zdhhc17	9.11E-08	-0.42132	0.199	0.413	0.002
Tmcc1	1.72E-06	-0.42188	0.285	0.497	0.037775
Hspa12b	7.25E-07	-0.42759	0.272	0.497	0.015915
Coro2b	9.91E-10	-0.43017	0.269	0.533	2.18E-05
Reps2	3.32E-07	-0.43051	0.171	0.365	0.007299
Nipal3	7.24E-07	-0.43143	0.332	0.557	0.015898
Cd2ap	2.28E-08	-0.43171	0.396	0.659	0.0005
Enho	2.33E-08	-0.43225	0.251	0.497	0.000511
Prdm1	1.78E-06	-0.43426	0.293	0.509	0.039035
Asap1	1.66E-06	-0.43471	0.461	0.713	0.036367
Rb1cc1	1.30E-07	-0.43545	0.391	0.623	0.002862
Hspb1	4.91E-07	-0.43552	0.513	0.737	0.010781
Rap1a	1.40E-06	-0.43692	0.63	0.85	0.030666

S1pr1	2.18E-06	-0.43732	0.552	0.784	0.047801
Hook3	7.14E-07	-0.43734	0.415	0.647	0.015673
Rasa1	4.02E-07	-0.43766	0.329	0.551	0.008824
Nbr1	2.64E-07	-0.43802	0.236	0.431	0.005798
Ctnnd1	7.09E-07	-0.43935	0.567	0.808	0.015565
Fmnl3	8.90E-07	-0.44368	0.244	0.449	0.01955
Tmbim1	2.03E-07	-0.44575	0.391	0.623	0.004449
Tln2	1.97E-06	-0.44794	0.381	0.605	0.043265
Stxbp1	1.96E-06	-0.44833	0.249	0.449	0.043086
Cdc42bpa	2.16E-06	-0.44866	0.482	0.731	0.04734
Hip1	1.53E-07	-0.45023	0.399	0.659	0.003364
Lhfp	1.08E-06	-0.45085	0.456	0.725	0.023699
Nbeal1	4.05E-08	-0.45243	0.345	0.605	0.000889
Sema3f	1.51E-06	-0.45478	0.492	0.749	0.033061
Afg1l	2.25E-07	-0.45619	0.218	0.431	0.004936
Rasgef1a	4.37E-07	-0.45666	0.37	0.623	0.009607
Fam219a	4.54E-07	-0.4571	0.337	0.587	0.009969
Qk	4.35E-07	-0.45811	0.731	0.898	0.009542
Inpp5a	5.10E-08	-0.45901	0.438	0.737	0.001119
Gm4258	3.00E-07	-0.45904	0.218	0.443	0.006582
Ppp2r3a	1.48E-06	-0.45953	0.194	0.377	0.032456
2610203C22Rik	1.32E-06	-0.46214	0.293	0.515	0.028928
Plpp1	1.23E-07	-0.46292	0.464	0.749	0.002707
Nck1	8.68E-09	-0.464	0.342	0.599	0.000191
Osbpl8	3.26E-08	-0.46614	0.459	0.737	0.000716
Tbx3	1.39E-07	-0.46718	0.536	0.778	0.003053
Rcsd1	2.71E-07	-0.46791	0.404	0.653	0.005943
Irs1	1.93E-06	-0.47125	0.215	0.401	0.042438
Dpysl3	9.75E-07	-0.47197	0.262	0.467	0.02142
Slco3a1	1.88E-08	-0.47239	0.417	0.683	0.000413
Cavin2	1.03E-06	-0.47361	0.593	0.814	0.022539
Mef2a	6.48E-08	-0.47366	0.614	0.88	0.001424
Ptk2	1.31E-08	-0.4762	0.5	0.778	0.000287
Klf2	4.02E-07	-0.47758	0.565	0.79	0.008825
Casz1	6.28E-07	-0.47826	0.505	0.743	0.013798
Myct1	2.78E-07	-0.47901	0.435	0.701	0.006106
Hexb	1.98E-07	-0.48054	0.492	0.76	0.004347
Impdh1	1.44E-07	-0.48276	0.358	0.593	0.003173
Tnfsf10	2.73E-09	-0.4828	0.332	0.611	5.99E-05
Zfhx3	2.24E-07	-0.48282	0.606	0.832	0.004929
Stim2	5.06E-07	-0.48518	0.412	0.647	0.011102

Ccnl2	3.23E-07	-0.48686	0.303	0.521	0.007096
Kat2b	9.83E-08	-0.48697	0.389	0.629	0.002159
Atp8a1	1.81E-08	-0.48807	0.772	0.922	0.000397
Krt80	1.31E-08	-0.48873	0.409	0.701	0.000287
Bdkrb2	1.05E-07	-0.48892	0.101	0.287	0.002315
Map7d1	1.34E-07	-0.4893	0.399	0.653	0.002953
Nhlrc2	1.69E-06	-0.4911	0.523	0.737	0.03706
Otud7b	6.79E-07	-0.4927	0.337	0.563	0.014915
Srgap2	9.49E-07	-0.49399	0.407	0.635	0.02083
Ahr	2.14E-07	-0.494	0.427	0.689	0.004702
Syne1	3.28E-07	-0.49427	0.508	0.79	0.007205
Igf1r	1.55E-07	-0.49591	0.409	0.683	0.003412
Itga2	1.23E-07	-0.49769	0.295	0.539	0.002691
Trerf1	1.67E-06	-0.49807	0.368	0.605	0.036708
Cdh5	4.72E-07	-0.49958	0.614	0.79	0.010363
Cyyr1	3.16E-07	-0.5	0.484	0.749	0.006931
Galnt18	3.11E-07	-0.50047	0.598	0.844	0.006822
Thbd	2.28E-07	-0.50274	0.552	0.784	0.005015
Itgb5	3.00E-08	-0.50448	0.365	0.635	0.000659
Mbd5	1.53E-06	-0.50557	0.402	0.605	0.03362
Marcks	1.23E-06	-0.50561	0.503	0.713	0.026949
Hecw2	6.66E-08	-0.50614	0.412	0.665	0.001463
Tspan13	3.17E-07	-0.50806	0.611	0.82	0.006953
Pik3cb	2.87E-08	-0.50854	0.368	0.629	0.00063
Klhl5	1.76E-07	-0.50873	0.396	0.635	0.003862
Stag2	6.00E-07	-0.50897	0.448	0.695	0.013176
Clec14a	2.07E-06	-0.50905	0.394	0.611	0.045417
Rin2	1.46E-08	-0.51036	0.415	0.683	0.00032
Cdkal1	1.65E-06	-0.51111	0.345	0.551	0.036125
Stag1	3.06E-07	-0.51413	0.505	0.754	0.006725
Dock4	6.11E-07	-0.51444	0.676	0.85	0.013419
Dennd1a	6.78E-07	-0.51486	0.383	0.635	0.014898
Kalrn	3.20E-08	-0.51532	0.508	0.749	0.000702
Pcdh1	1.10E-07	-0.51563	0.482	0.719	0.002421
Ptprg	1.02E-06	-0.517	0.697	0.868	0.022343
Psmd14	3.22E-07	-0.5174	0.238	0.443	0.007081
Mcc	6.41E-09	-0.51767	0.541	0.844	0.000141
Foxf1	5.85E-08	-0.51856	0.446	0.737	0.001285
Nrp1	2.13E-08	-0.52424	0.72	0.892	0.000467
Tjp2	2.48E-09	-0.52507	0.521	0.808	5.44E-05
Emp1	4.55E-07	-0.52539	0.469	0.731	0.010003

Meis1	2.14E-07	-0.5256	0.474	0.737	0.004696
Tmod2	1.26E-08	-0.52672	0.35	0.611	0.000277
Pmp22	9.74E-09	-0.5268	0.56	0.826	0.000214
Prickle1	2.60E-07	-0.52686	0.42	0.659	0.005711
Scn7a	1.76E-07	-0.5279	0.565	0.766	0.003869
Phactr1	4.33E-07	-0.52914	0.42	0.647	0.009499
Id1	4.45E-08	-0.53022	0.368	0.623	0.000977
Ptpn4	1.27E-06	-0.53117	0.477	0.701	0.027835
Fgf1	1.27E-07	-0.53203	0.376	0.611	0.00279
Klc1	3.12E-09	-0.53378	0.282	0.551	6.86E-05
Afap1l1	6.45E-07	-0.53793	0.503	0.749	0.014168
Tjp1	1.62E-09	-0.53973	0.552	0.802	3.56E-05
Lysmd2	2.81E-07	-0.54216	0.22	0.431	0.006166
Fry	5.95E-08	-0.54366	0.42	0.671	0.001307
Nckap1	1.15E-09	-0.5441	0.433	0.695	2.52E-05
Ankrd33b	2.83E-08	-0.54437	0.466	0.725	0.000621
Tmcc3	1.13E-08	-0.54441	0.5	0.76	0.000248
Sptbn1	4.21E-10	-0.5452	0.767	0.94	9.25E-06
Lifr	4.60E-09	-0.54551	0.313	0.563	0.000101
Mapk6	6.99E-09	-0.54648	0.368	0.659	0.000153
Mtus1	7.61E-09	-0.54697	0.448	0.707	0.000167
Agfg1	7.73E-09	-0.54725	0.547	0.814	0.00017
Ehbp1	1.85E-07	-0.54811	0.316	0.539	0.004064
Sash1	5.68E-08	-0.54925	0.495	0.766	0.001248
Gmds	6.51E-09	-0.54961	0.215	0.461	0.000143
Rbfox2	1.13E-07	-0.55094	0.477	0.737	0.002483
Rbms1	2.99E-08	-0.55185	0.728	0.91	0.000657
Prx	1.57E-08	-0.55293	0.482	0.749	0.000346
Nfib	1.97E-08	-0.55306	0.681	0.898	0.000432
Emp2	1.12E-08	-0.55391	0.741	0.916	0.000247
Frmd6	3.70E-11	-0.55481	0.417	0.737	8.13E-07
Itpkb	6.00E-09	-0.55565	0.49	0.784	0.000132
Gnao1	2.56E-10	-0.55665	0.363	0.671	5.62E-06
Gab1	1.75E-06	-0.5569	0.352	0.551	0.038454
Pecam1	6.21E-08	-0.5586	0.601	0.802	0.001364
Apln	1.75E-07	-0.56101	0.342	0.605	0.003844
Stard13	2.19E-08	-0.5616	0.427	0.689	0.000481
Arhgef12	1.49E-09	-0.56395	0.681	0.904	3.28E-05
Ssh2	4.02E-07	-0.56435	0.539	0.743	0.008821
Zfp641	4.90E-07	-0.5654	0.197	0.389	0.010756
Car4	7.79E-08	-0.56981	0.57	0.802	0.001712

Fmnl2	1.12E-07	-0.57107	0.554	0.766	0.002461
Prkd1	2.10E-09	-0.57176	0.282	0.551	4.62E-05
Acvrl1	4.23E-07	-0.57267	0.552	0.766	0.009296
Piezo2	4.64E-08	-0.57275	0.526	0.772	0.001018
Chst1	2.95E-10	-0.57317	0.337	0.641	6.48E-06
Phactr2	7.00E-07	-0.57445	0.381	0.593	0.015366
Csgalnact1	8.33E-09	-0.57995	0.466	0.76	0.000183
Slco2a1	2.74E-08	-0.58225	0.648	0.838	0.000601
Rgs12	2.95E-07	-0.58327	0.503	0.713	0.006484
Gnaq	1.30E-08	-0.58404	0.622	0.868	0.000285
Npepps	9.42E-09	-0.58498	0.345	0.593	0.000207
Arhgap31	1.61E-08	-0.58559	0.611	0.808	0.000354
Gata2	6.65E-10	-0.58564	0.352	0.647	1.46E-05
Zfp715	1.07E-09	-0.58591	0.109	0.317	2.34E-05
Epas1	9.62E-10	-0.5868	0.684	0.91	2.11E-05
Ntn4	4.68E-10	-0.58738	0.339	0.635	1.03E-05
Fibin	3.70E-08	-0.58914	0.433	0.701	0.000813
Ace	4.26E-09	-0.59074	0.451	0.719	9.35E-05
Ptpre	3.04E-09	-0.59306	0.267	0.515	6.67E-05
Arhgef3	2.64E-08	-0.59331	0.58	0.808	0.00058
Hpgd	2.09E-06	-0.5958	0.497	0.713	0.045875
Bmpr2	1.09E-09	-0.59797	0.692	0.886	2.40E-05
Snrk	9.67E-09	-0.59931	0.451	0.713	0.000212
Sema6a	3.78E-08	-0.60033	0.523	0.766	0.000829
Ehd4	2.45E-09	-0.60147	0.663	0.892	5.38E-05
Hdac7	1.96E-09	-0.60516	0.409	0.671	4.29E-05
Dennd3	4.54E-08	-0.60647	0.313	0.551	0.000997
Kdr	2.62E-09	-0.60767	0.598	0.82	5.75E-05
Clic5	3.89E-10	-0.6099	0.637	0.892	8.53E-06
App	1.48E-12	-0.61082	0.751	0.958	3.25E-08
Nav1	1.13E-08	-0.61231	0.492	0.737	0.000249
Egflam	2.05E-08	-0.61553	0.378	0.635	0.000451
Cyp4b1	9.77E-09	-0.61857	0.601	0.82	0.000215
Pakap.1	6.17E-08	-0.62465	0.464	0.707	0.001355
Pde9a	1.86E-10	-0.62582	0.189	0.443	4.08E-06
Clec1a	1.22E-08	-0.62758	0.433	0.731	0.000268
Klf12	3.58E-09	-0.62974	0.446	0.743	7.86E-05
Itga3	6.64E-12	-0.63039	0.409	0.719	1.46E-07
Ampd3	9.76E-08	-0.63055	0.295	0.539	0.002143
Klf7	1.11E-09	-0.63396	0.604	0.862	2.44E-05
Stard8	2.57E-09	-0.63432	0.427	0.689	5.64E-05

Fgd5	1.48E-09	-0.63674	0.37	0.659	3.25E-05
Aqp1	7.57E-08	-0.63806	0.466	0.737	0.001663
Prkce	1.05E-08	-0.6402	0.668	0.856	0.00023
Snx13	2.27E-10	-0.64183	0.35	0.605	4.98E-06
Ptprk	2.68E-07	-0.64252	0.575	0.784	0.005879
Samd4	9.38E-09	-0.64368	0.378	0.623	0.000206
Cdk14	1.12E-10	-0.64503	0.627	0.856	2.47E-06
Mapt	7.78E-11	-0.64633	0.425	0.743	1.71E-06
A330023F24Rik	8.24E-10	-0.64805	0.345	0.635	1.81E-05
Ednrb	1.91E-09	-0.65048	0.51	0.766	4.20E-05
Tspan18	2.98E-08	-0.65097	0.484	0.725	0.000655
Ghr	3.70E-08	-0.65303	0.505	0.76	0.000812
Smarca2	4.31E-11	-0.65343	0.645	0.886	9.46E-07
Tspan9	1.30E-10	-0.65813	0.534	0.814	2.86E-06
Adgrf5	3.20E-11	-0.6583	0.718	0.916	7.03E-07
Ankrd44	2.69E-08	-0.66137	0.567	0.796	0.000591
Ctdspl	4.07E-09	-0.66839	0.409	0.653	8.94E-05
Oxr1	4.23E-08	-0.66857	0.541	0.784	0.000929
Fnbp1l	4.13E-10	-0.67395	0.456	0.713	9.06E-06
Lmo7	1.03E-09	-0.67536	0.547	0.838	2.27E-05
Dach1	1.21E-08	-0.67636	0.544	0.784	0.000265
Sppl3	2.40E-09	-0.67841	0.461	0.707	5.27E-05
Zbtb46	1.37E-07	-0.6795	0.313	0.545	0.003006
Adgrl2	1.07E-09	-0.67964	0.601	0.868	2.36E-05
Psd3	3.41E-10	-0.68141	0.63	0.886	7.48E-06
Fendrr	9.13E-09	-0.68352	0.412	0.671	0.000201
Podxl	3.95E-08	-0.68405	0.495	0.737	0.000868
Gm42418	4.61E-07	-0.6876	1	1	0.010132
Raph1	7.43E-11	-0.6882	0.451	0.737	1.63E-06
Smurf2	7.80E-12	-0.68989	0.549	0.838	1.71E-07
Kitl	2.81E-10	-0.69063	0.632	0.856	6.18E-06
Slc16a9	7.26E-10	-0.69372	0.332	0.599	1.59E-05
Dcbld1	4.97E-09	-0.6943	0.288	0.527	0.000109
Flt1	1.00E-09	-0.69464	0.477	0.731	2.20E-05
Kif26b	8.20E-12	-0.70035	0.179	0.449	1.80E-07
Arhgap29	4.16E-11	-0.70196	0.49	0.808	9.14E-07
Mertk	1.69E-13	-0.7021	0.358	0.695	3.72E-09
Arap3	8.82E-12	-0.71183	0.386	0.695	1.94E-07
Zfp697	1.31E-12	-0.71799	0.321	0.653	2.88E-08
Plxna2	5.49E-09	-0.72083	0.453	0.701	0.000121
Ston2	3.89E-08	-0.72259	0.339	0.605	0.000855

Cyth3	9.76E-12	-0.72275	0.562	0.844	2.14E-07
Sgip1	2.62E-09	-0.72471	0.425	0.695	5.76E-05
Tbx3os1	1.79E-08	-0.72501	0.376	0.641	0.000393
Stxbp6	9.79E-11	-0.73143	0.469	0.766	2.15E-06
Stard9	8.88E-11	-0.73217	0.402	0.683	1.95E-06
Aff3	5.68E-11	-0.7414	0.622	0.796	1.25E-06
Camk1d	1.58E-06	-0.74195	0.733	0.952	0.034587
Fcho2	7.67E-11	-0.74385	0.518	0.796	1.68E-06
Fmo1	4.83E-11	-0.76067	0.503	0.784	1.06E-06
Myzap	1.90E-10	-0.76372	0.453	0.725	4.18E-06
Calcrl	2.05E-11	-0.76486	0.674	0.838	4.51E-07
Carmil1	1.33E-11	-0.7674	0.487	0.778	2.93E-07
Zbtb16	3.63E-07	-0.77124	0.329	0.521	0.007967
D630045J12Rik	1.36E-09	-0.77145	0.28	0.527	2.99E-05
Lmntd1	1.27E-10	-0.77163	0.383	0.671	2.79E-06
Tmeff2	1.83E-09	-0.77784	0.396	0.677	4.01E-05
Msi2	3.75E-12	-0.79253	0.653	0.88	8.24E-08
Rasal2	5.52E-11	-0.79339	0.544	0.802	1.21E-06
Tspan7	1.95E-09	-0.79791	0.547	0.79	4.27E-05
Cep85l	1.28E-13	-0.80268	0.404	0.737	2.81E-09
Acap2	3.96E-12	-0.80828	0.5	0.82	8.69E-08
Vav3	1.36E-10	-0.80916	0.505	0.766	2.98E-06
Edil3	6.09E-13	-0.82107	0.415	0.737	1.34E-08
Prickle2	3.74E-11	-0.83562	0.666	0.844	8.22E-07
Gm30382	1.65E-11	-0.84316	0.365	0.695	3.63E-07
Rgs6	1.10E-10	-0.85352	0.518	0.772	2.43E-06
Auts2	6.10E-12	-0.87668	0.378	0.695	1.34E-07
Pdgfd	7.85E-13	-0.87841	0.402	0.719	1.72E-08
Nectin3	1.89E-17	-0.87891	0.469	0.838	4.15E-13
Ccdc85a	4.59E-10	-0.88414	0.326	0.605	1.01E-05
Gm29865	6.58E-07	-0.91604	0.047	0.174	0.014451
Tmtc1	3.19E-14	-0.93043	0.394	0.701	7.01E-10
Smad6	1.45E-12	-0.97293	0.453	0.725	3.19E-08

<u>Supplemental Table 2</u>: Differences in protein expression within regions of interest (ROIs) in IPF vs. control (n = 12 ROIs)

Targets	Log_2	*p* value	Adjusted *p* value	-log10 *p* value	-log10 adjusted *p* value
CTLA4	5.016961671	4.82739E-10	1.78613E-08	9.316287914	7.74808619
SMA	3.733023038	2.10868E-13	1.56042E-11	12.67599003	10.80675831
CD56	1.056955924	0.000105425	0.000557249	3.97705477	3.253951085
PD-1	0.887427724	0.002341709	0.004912013	2.630467132	2.30874046
GAPDH	0.855982851	0.006524526	0.012379869	2.18545106	1.907283947
Phospho-AKT (phospho T308)	0.618599087	0.048941807	0.071013603	1.310319997	1.148658453
CD4	0.538556041	0.030698614	0.04732703	1.512881235	1.324890753
CD3	0.514185138	0.082071248	0.110423134	1.08580896	0.95693993
CD20	0.434416631	0.162735785	0.200707468	0.788516937	0.697436467
S100B	0.340823553	0.389579871	0.436801673	0.409403491	0.359715707
PTEN	0.285990142	0.277616519	0.324696324	0.556554695	0.488522629
Ki-67	0.271755303	0.326226182	0.371395961	0.486481186	0.430162823
CD11c	0.2016193	0.438573143	0.484394218	0.357957966	0.314801049
CD163	0.134871689	0.684085418	0.712990436	0.164889667	0.146916296
CD45	0.12761867	0.66147493	0.699273498	0.179486611	0.155352931
GZMB	0.125761775	0.592619742	0.635563201	0.227223885	0.196841256
INPP4B	0.015975727	0.947124546	0.947124546	0.023592908	0.023592908
CD27	-0.061189867	0.793328827	0.804196345	0.100546765	0.094637905
Beta-2-microglobulin	-0.087855146	0.694738815	0.714037115	0.158178436	0.146279213
PD-L1	-0.15691708	0.591005596	0.635563201	0.228408407	0.196841256
BCL6	-0.300338026	0.237866463	0.288559316	0.623666785	0.5397649
PD-L2	-0.308077571	0.241898572	0.28871765	0.616366696	0.539526665
p53	-0.355587018	0.150287094	0.188495678	0.823078312	0.724698604
CD14	-0.357813376	0.138501624	0.176708968	0.858545135	0.752741409
GZMA	-0.373292592	0.280818442	0.324696324	0.551574374	0.488522629
BAD	-0.399365525	0.135920661	0.176458402	0.866714523	0.753357658
CD95/Fas	-0.424881696	0.073561013	0.10468298	1.133352299	0.980123922
ICOS	-0.429894907	0.076926211	0.1054174	1.11392566	0.9770877
BIM	-0.437439178	0.089973296	0.118893283	1.045886372	0.924842679
CD45RO	-0.49438767	0.020722473	0.033336152	1.683558421	1.477084533
Phospho-p38 MAPK (T180/Y182)	-0.510976142	0.076086564	0.1054174	1.118692027	0.9770877
CD127	-0.52374526	0.025085264	0.039495948	1.600581319	1.403447457
Pan-AKT	-0.558623749	0.038186683	0.057669684	1.41808807	1.23905243
CD25	-0.599308834	0.01457647	0.023970194	1.836347651	1.620328445

Cleaved Caspase 9	-0.649480318	0.003867927	0.007735854	2.412521748	2.111491752
S6	-0.653359406	0.004735426	0.009221619	2.324640954	2.035192831
PARP	-0.725495975	0.00237775	0.004912013	2.623833822	2.30874046
pan-RAS	-0.759806085	0.000635656	0.001959938	3.196778042	2.707757564
Histone H3	-0.761310773	0.008465175	0.014914832	2.072364064	1.826381635
BRAF	-0.784580625	0.012176336	0.020478383	1.914483391	1.688704348
PR	-0.805453326	0.002361534	0.004912013	2.626805797	2.30874046
Phospho-MEK1 (S217/S221)	-0.821048428	0.01009786	0.017377712	1.995770671	1.760007407
CD8	-0.824132229	0.007498904	0.013872973	2.125002184	1.857830456
CD40	-0.846303021	0.001519013	0.00362603	2.818438579	2.440568553
PLCG1	-0.896699931	0.008256863	0.014902631	2.083184926	1.826737063
NY-ESO-1	-0.906553804	0.000998641	0.002548256	3.000590688	2.593756966
Bcl-2	-0.924585492	0.000630262	0.001959938	3.200478644	2.707757564
Fibronectin	-0.943253072	0.000827749	0.002450136	3.082101522	2.610809811
MET	-1.022837257	0.000137687	0.0006368	3.861108346	3.195996609
ER-alpha	-1.025372947	0.000882818	0.002461656	3.054128899	2.608772701
CD66b	-1.025796273	0.043205722	0.063944468	1.364458737	1.194197021
Phospho-Tuberin (T1462)	-1.048985888	0.002389628	0.004912013	2.621669679	2.30874046
Phospho-c-RAF (S338)	-1.09054349	0.000898172	0.002461656	3.046640657	2.608772701
MART1	-1.128606988	0.000590836	0.001959938	3.228532973	2.707757564
Her2	-1.166655997	3.5175E-05	0.000260295	4.453765902	3.584534182
FAP-alpha	-1.20904668	5.39379E-05	0.000358514	4.268106087	3.445493774
Phospho-GSK3A (S21)/Phospho-GSK3B (S9)	-1.230812025	0.000261912	0.001045819	3.581844826	2.980543648
FOXP3	-1.240280195	0.002341282	0.004912013	2.630546199	2.30874046
CD80	-1.242664001	0.000268521	0.001045819	3.571021767	2.980543648
HLA-DR	-1.328104854	0.000938102	0.002479271	3.027749731	2.605676042
BCLXL	-1.408107273	0.000119781	0.000590921	3.921611166	3.228470706
Phospho-AKT1 (S473)	-1.414056112	8.62523E-05	0.000490975	4.064229102	3.308940734
Phospho-GSK3B (S9)	-1.423087145	0.000530843	0.001870589	3.275033984	2.728021559
p44/42 MAPK ERK1/2	-1.48413735	0.000230646	0.001003986	3.637054944	2.998272145
CD68	-1.500054245	0.000324535	0.001200778	3.488739022	2.920537298
CD44	-1.563017605	5.0224E-06	6.19429E-05	5.299088659	4.208008189
EGFR	-1.585486552	3.56517E-06	5.27645E-05	5.447920279	4.277658563

Phospho-p44/42 MAPK ERK1/2 (T202/Y204)	-1.677844514	0.001429809	0.003526863	2.844721898	2.452611433
Phospho-p90 RSK (T359/S363)	-1.82956498	6.70847E-06	7.09181E-05	5.173376762	4.149243083
Phospho-PRAS40 (T246)	-1.84994348	2.41268E-05	0.000198376	4.617501133	3.702511923
PanCk	-1.960132473	5.81374E-05	0.000358514	4.235544248	3.445493774
CD34	-2.349532733	8.72852E-06	8.07388E-05	5.059059508	4.092917776
EpCAM	-2.498362336	5.20619E-08	9.63146E-07	7.283479776	6.016308048
Phospho-JNK (T183/Y185)	-2.517583637	2.3511E-08	5.79937E-07	7.628729337	6.236618872
